# Tidal Trapping and Its Effect on Salinity Dispersion in Well-Mixed Estuaries Revisited

**DOI:** 10.1007/s12237-025-01579-0

**Published:** 2025-07-30

**Authors:** Daan van Keulen, Wouter M. Kranenburg, Antonius J. F. Hoitink

**Affiliations:** 1 Department of Environmental Sciences, Wageningen University, Wageningen, The Netherlands; 2 Department of Hydraulic Engineering, Delft University of Technology, Delft, The Netherlands; 3 Deltares, Delft, The Netherlands

**Keywords:** Tidal dispersion, Tidal trapping, Channel-trap exchange, Dead-end side channels

## Abstract

In well-mixed estuaries, the up-estuary salt flux is often dominated by tidal dispersion mechanisms, including tidal trapping. Tidal trapping involves volumes of water being temporarily trapped in dead zones or side channels adjacent to the main channel and released later in the tidal cycle, which causes an additional up-estuary salt flux. Tidal trapping can result from a diffusive exchange between a channel and a trap, or from filling and emptying of the trap by a tidal flow that is ahead in phase compared to the flow in the main channel (advective out-of-phase exchange). This study revisits the dispersive contribution from tidal trapping in a single dead-end side channel using an idealized numerical model. The results indicate that advective out-of-phase exchange yields the largest additional salt flux for the largest realistic velocity phase difference of 90$^\circ$. Mixing of the trapped salinity field enhances the dispersive effect for small velocity phase differences. A continuous diffusive channel-trap exchange also enhances the dispersive trap effect when the velocity phase difference is small, but can dampen it when the phase difference is large. We demonstrate that the effect of a trap is twofold: firstly, channel-trap exchange alters the salinity field and introduces an additional salt flux in the main channel over a distance equal to the tidal excursion length; secondly, the altered salinity gradients are advected in both up- and down-estuary direction, influencing the tidal salt flux over twice the excursion length.

## Introduction

In estuaries, there is a continuous competition between flushing of salt water by freshwater river discharge and up−estuary salt transporting mechanisms. In a partially or strongly stratified system, the up−estuary salt flux is predominantly driven by gravitational circulation (Hansen & Rattray Jr, 1965), which can be enhanced by asymmetry in mixing between flood and ebb (Jay & Musiak, 1996; Geyer & MacCready, 2014). In well-mixed and generally shorter estuaries, the up-estuary salt flux often results from tidal dispersion mechanisms. Tidal dispersion includes phenomenon like jet-sink exchange (Stommel & Former, 1952), which typically occur near the mouth of estuarine systems. Here, the outflow exhibits jet-like characteristics, while the inflow during flood is more evenly distributed, resulting in import of non-native (saline) water. Another widely studied tidal dispersion mechanism is tidal trapping, where volumes of water are temporarily trapped in dead zones or side branches and re-discharged later in the tidal cycle (Okubo, 1973; Dronkers, 1978; MacVean & Stacey, 2011). Often, tidal dispersion mechanisms are introduced by geometric features like channel constrictions, shoals, and meanders (Garcia & Geyer, 2022), though tidal dispersion can also be introduced in straight channels by a phase shift of the salinity signal due to river discharge (Dijkstra et al., 2022).

To distinguish various transport mechanisms, the tidally averaged salt flux through a cross-section can be decomposed (Fischer et al., 1979; Lerczak et al., 2006). In a salt flux decomposition, tidal dispersion mechanisms are reflected in the correlation between the tidally varying demeaned velocity and salinity signals. This correlation reflects an up-estuary salt flux when the phase difference is less than in quadrature. Dronkers and Van de Kreeke (1986) demonstrated that in a Eulerian framework, the correlation-related salt flux is the result of vertical or lateral exchange occurring elsewhere within the tidal excursion and is, therefore, termed non-local salt flux. Recently, Garcia and Geyer (2022) applied the decomposition of Dronkers and Van de Kreeke (1986) to study the origin of the tidal salt flux in the North River, linking the observed Eulerian salt flux to specific geometric features.

This study focuses on tidal trapping due to diffusive and advective exchange between a channel and a trap and their effect on tidal dispersion. As mentioned above, tidal trapping results from the temporal storage in dead zones or side branches, referred to as traps. A net (additional) salt flux in the main channel occurs because the exchange between the channel and the trap causes volumes of water to be temporarily stored and then released at different times in the tidal cycle, introducing an up-estuary salt flux. Exchange with a trap may occur through lateral exchange mechanisms including those introduced by eddies (Dronkers, 1978; Geyer & Signell, 1992) or density currents set up by density differences between the channel and the trap (Abraham et al., 1986; Ralston & Stacey, 2005; Giddings et al., 2012; Garcia et al., 2022). These processes rely on spatial variations in the flow and salinity fields at the trap entrance, which, in a cross-sectional-averaged sense, are diffusive in nature (hereafter referred to as diffusive exchange). A net salt flux can also result from the velocity phase difference between the cross-sectional averaged current in the channel and in the trap (Dronkers, 1978; MacVean & Stacey, 2011). This phase difference arises because the wave-type, which labels the phase relationship between variations in water level and the flow, typically differs between the channel and the trap. In the trap, which acts as a short basin, currents directly respond to water level variations, with high and low water coinciding with slack water (i.e. water level variations are in quadrature with the currents). In contrast, due to inertial effects, the main channel typically exhibits a phase difference between high or low water levels and the corresponding moments of slack water (Friedrichs, 2010). In the most extreme case of a progressive wave, this can cause the currents to be in phase with the water level variations. The velocity phase difference between the channel and the trap causes a relative displacement between the parcels that are trapped and those that remain in the main channel (Dronkers, 1978). This introduces a longitudinal dispersion mechanism, referred to as advective out-of-phase exchange by Garcia et al. (2022).

Several authors have derived an analytical expression to quantify the effect of tidal trapping, assuming either diffusive or advective out-of-phase exchange and evaluating a finite or continuous trap. For diffusive channel-trap exchange, Okubo (1973) derived an analytical model intended to quantify the influence of shoreline irregularities, assuming a continuous lateral trap that exchanges continuously with the main channel. Through the concentration moment analysis (Aris, 1956), a solution for the effective dispersion of salt in the main channel, $K_\text {eff}$, is obtained:

1 $$\begin{aligned} K_\text {eff,Okubo} = \underbrace{\frac{r\hat{U_c}^2 }{\omega }\biggr (\frac{\omega /k}{2(1+r)^2(1+r+\omega /k)}\biggl )}_{K_\text {trp,Okubo}} + \frac{K_b}{1+r} , \end{aligned}$$

where $r=B_t/B_c$ is the trap to channel width ratio, *k* is the exchange-rate coefficient with $k^{-1}$ being the residence time, $\hat{U_c}$ is the velocity amplitude in the main channel, $\omega = 2\pi /T$ is the radian frequency (with *T* the tidal period), and $K_b$ is the background dispersion coefficient (encompassing all dispersive processes other than tidal trapping). In Eq. 1, the effective dispersion results from the first term, $K_\text {trap,Okubo}$, which represents the additional dispersive effect caused by tidal trapping, and the second term, which represents a reduction of the background dispersion caused by trapped particles not being subjected to dispersion in the main channel. Although, strictly speaking, Eq. 1 is only applicable to a continuous lateral trap and diffusive exchange, it is often used to estimate the contribution of finite traps where the exchange is driven by advective out-of-phase exchange (MacVean & Stacey, 2011; Garcia et al., 2022).

MacVean and Stacey (2011) highlighted that Eq. 1 does not capture the dynamics of advective out-of-phase exchange. Assuming a source-sink term driven by advective exchange that is out-of-phase with the main channel and a salinity at the trap entrance independent of the salinity in the main channel, they used the concentration moment analysis to derive an alternative expression for the additional dispersion:

2 $$\begin{aligned} K_\text {eff,MacVean} \!=\! \underbrace{\frac{\epsilon \hat{U_c}^2 }{\omega } \bigl ( \sin \alpha \cos \alpha \Bigl ( \frac{3 \cos \alpha \!+\! 32 \cos \alpha }{12\pi } \Bigr ) \biggl )}_{K_\text {trp,MacVean}} \!+\! K_b, \end{aligned}$$

with $\alpha$ the velocity phase difference between the flow in the trap and in the channel, and $\epsilon = (A_t\hat{U}_t \hat{S}_t)/(A_c\hat{U_c} \hat{S})$ representing the salt flux through the trap entrance relative to the salt flux in the channel, $\hat{U}_t$ the velocity amplitude in the trap, $A_t$ is the cross-sectional area of the trap, and $\hat{S}_t$ and $\hat{S}$ are the salinity amplitudes in the trap and the main channel, respectively. Again, the first term represents the additional dispersive effect caused by tidal trapping ($K_\text {trap,MacVean}$), and the second term represents the effect of the background dispersion. According to Eq. 2, the trap effect is greatest when $\alpha \approx \frac{1}{4}\pi$, and no net effect is expected for the largest physically realistic phase difference of $\frac{1}{2}\pi$. A distinct difference with Eq. 1 is that it is derived under the assumption of a trap existing over a limited stretch, which is short compared to the tidal excursion length.

Dronkers (1978) derived analytical expressions for both continuous traps and finite traps. Instead of using a concentration moment analysis, he adopted a Lagrangian approach to quantify the additional salt flux due to tidal trapping. Furthermore, this work clearly outlines why the additional salt flux is variable in space for a trap that is short compared to the excursion length, but becomes constant for a continuous lateral trap. For a continuous lateral trap subject to out-of-phase exchange, Dronkers (1978) obtained the following expression:

3 $$\begin{aligned} K_\text {trp,Dronkers} = \frac{2}{3} \frac{ H_t B_t}{A_c} \frac{L_t^2}{T} \sin ^2\alpha , \end{aligned}$$

where $L_t$ is the tidal excursion length and $H_t$ is the depth in the trap. In the case where the depths in the channel and trap are equal, Eq. 3 closely resembles Eq. 1. Then, the geometry ratio reduces to *r*, and the dimensional group is rewritten as $\frac{L_t^2}{T} \sim \frac{\hat{U}_c^2}{\omega }$. A notable difference between Eq. 3 from Dronkers (1978) and Eq. 2 from MacVean and Stacey (2011) is that in Eq. 3, the additional dispersion ($K_\text {trp}$) increases with the velocity phase difference and continues to increase up to $\alpha = \frac{1}{2}\pi$, while Eq. 2 predicts a decrease for $\alpha > \frac{1}{4}$. Thus, the formulation by Dronkers (1978) suggests that the trap effect is strongest at the maximum physical phase difference ($\alpha =\frac{1}{2}\pi$), which stands in contrast with the findings of MacVean and Stacey (2011).

Hence, various analytical frameworks exist to quantify the dispersion resulting from tidal trapping, but these frameworks differ significantly in how the effect of the trap depends on the phase difference between the velocity in the trap and in the channel. Furthermore, the existing analytical frameworks typically assume either pure diffusive or advective out-of-phase exchange, whereas in reality, both mechanisms can occur simultaneously, an aspect that so far has not been studied. Additionally, for traps with entrance widths much smaller than the tidal excursion (e.g. side channels), insight is lacking into the spatial variability of the additional salt flux with distance from the trap resulting from the channel-trap exchange (either from diffusive or advective out-of-phase exchange).

The aims of this study are to understand the dependence of the additional salt flux on the velocity phase difference, to study and quantify the additional salt flux when the channel-trap exchange occurs through both dispersive and advective out-of-phase exchange, and to establish the spatial variability of the additional salt flux for tidal trapping introduced by a dead-end side channel. To achieve this, we numerically evaluate tidal trapping induced by a dead-end side channel using an idealized 1D finite-volume model incorporating local sources and sinks (Section “Methods”). Subsequently, parameter dependency tests are performed to examine the impacts of alternative channel-trap exchange mechanisms on the resulting additional salt flux in the main channel over a single tidal cycle, achieved by perturbing a reference salinity field (Section “Results”). In the discussion (Section “Discussion”), we compare the numerical findings with the existing analytical frameworks. In addition, we discuss the validity of expressing the additional salt flux in terms of dispersion coefficients. We then examine the additional tidal salt flux under equilibrium conditions, which, at equilibrium, results not only from direct channel-trap exchange. Finally, conclusions are drawn in Section “Conclusions”.

**Fig. 1:**
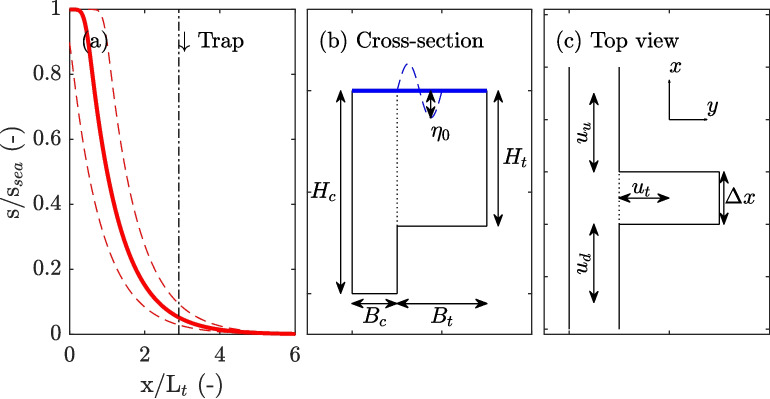
Overview of the model setup and symbols characterizing the dimensions of the trap. **a** Normalized subtidal salt curve used for the model initialization in Section “Results”, where the distance *x* is normalized by the tidal excursion length $L_t$. Dashed lines indicate the salinity at low water slack (LWS) and high water slack (HWS). **b** Cross-section over the trap and channel, with symbols for dimensions. Note that the horizontal and vertical dimensions are not drawn to scale. **c** Top view of the trap

## Methods

### Model Set-up

#### The Salt Balance

To investigate various scenarios and different types of channel-trap exchange, we use a simplified one-dimensional finite-volume model, which ensures mass conservation, to calculate the cross-sectional averaged salinity in the main channel. The basic salt-balance equation considered is given by

4a $$\begin{aligned} \frac{\partial s}{ \partial t} + \frac{\partial }{\partial x}\biggl ( su -K_b\frac{\partial s}{ \partial x} \biggr )&= \frac{I(x)}{A_c},\end{aligned}$$

4b $$\begin{aligned} s(0,t)&= s_{sea}, \end{aligned}$$

4c $$\begin{aligned} s(L_d,t)&= s_{riv}, \end{aligned}$$

with *s* representing the salinity in the main channel, *u* the main channel velocity (see Section “The Current Velocity in the Main Channel”), $K_b$ a constant background diffusion coefficient for all processes other than tidal trapping, $A_c$ a constant cross-sectional area for the main channel, and *I*(*x*) a source-sink term that represents the salt flux from a local trap in the model domain (see Section “Formulations for Channel-Trap Exchange”). Herein, the geometry of the main channel is assumed to be straight with a rectangular cross-section, but a spatially varying cross-sectional could be described. Boundary conditions are specified at the up-estuary and down-estuary ends with constant salinity values of $s_{riv} = 0$ and $s_{sea} = 35$, respectively, where $L_d$ denotes the length of the model domain.

For the initial salinity field, an exponentially decaying profile is prescribed:

5 $$\begin{aligned} s(x,0) = s_{sea} e^{\biggl (-\frac{u_r}{K_b}x \biggr )}, \end{aligned}$$

where $u_r$ is the velocity associated with the river discharge. This profile represents the equilibrium state if *u* were constant and equal to $u_r$. The associated time-scales are defined as $T_A = L_s/u_r$ for flushing and $T_D = L_s^2/K_b$ for the dispersive time-scale, where $L_s$ represents the salt intrusion length. Thus, different combinations of $u_r$ and $K_b$ can result in identical salinity distributions, but will differ in the corresponding adjustment time-scales. Adding the tidal movement to this system introduces an additional tidal salt flux.

#### The Current Velocity in the Main Channel

To examine the effect of velocity phase differences between the channel and the trap on the salinity profile and tidal dispersion, we consider a simplified flow field with a constant along-channel current velocity amplitude and phasing both upstream and downstream of the trap, with their difference influenced solely by the trap to ensure mass conservation. The system’s time origin ($t=0$) is set at low water, assuming that the trap’s current resembles a standing wave, so that the time origin coincides with low water slack in the trap. Tidal variations in the cross-sectional area $A_c$ are neglected. For the downstream stretch, we impose $u = \hat{U}_{c,d}\sin (\omega t - \alpha _d)$, which is used to calculate the up-estuary velocity amplitude and phasing based on the trap properties and mass conservation. Specifically, the amplitude and phase at the up-estuary cell are calculated from the down-estuary velocity amplitude $\hat{U}_{c,d}$, phase $\alpha _{d}$, and the trap’s discharge amplitude $\hat{q}_t$:

6 $$\begin{aligned} A_c \hat{U}_{c,d} \sin (\omega t - \alpha _d) - A_c \hat{U}_{c,u} \sin (\omega t - \alpha _u) - \Delta x \hat{q}_t \sin (\omega t) = 0. \end{aligned}$$

Rewriting Eq. 6 and expressing the amplitudes and phase in Fourier coefficients and evaluating the equation at $t = 0$ and $t = \alpha _d/\omega$ allow to express the up-estuary velocity amplitude $\hat{U}_{c,u}$ and phasing $\alpha _u$. The phasing downstream of the trap may vary between $0^\circ$ and $90^\circ$. We acknowledge that a fixed phase with a constant current velocity amplitude is inconsistent with fully realistic estuarine tidal properties, as described by Friedrichs (2010). However, it allows for a systematic evaluation of the effect of the velocity phase difference between the main channel and the trap. Given the discharge amplitude $\hat{q}_t= B_t \eta _0\omega$ at the trap entrance, the mean current within the trap, $u_t$, is given by

7 $$\begin{aligned} u_t= \frac{\eta _0\omega }{H_t -\eta _0 \cos (wt)}( B_t -y) \sin (\omega t), \end{aligned}$$

where *y* is the distance from the trap entrance, $\eta _0$ is the tidal amplitude, $H_t$ is the mean water depth in the trap, and $B_t$ is the width of the trap (Fig. 1). Equation 7 is used for the more complex source/sink term definitions, which require numerical evaluation of the salinity field within the trap.

**Fig. 2:**
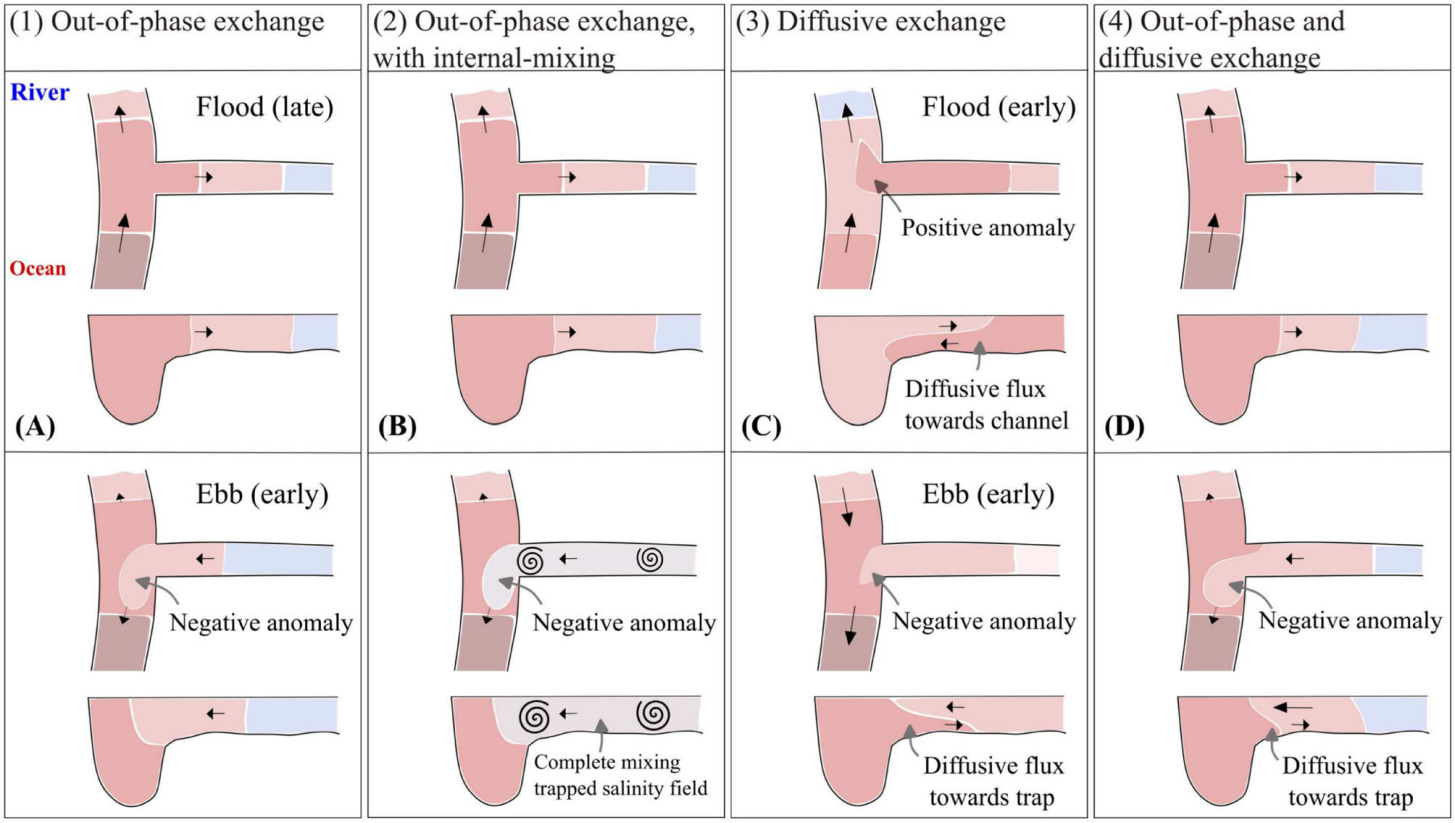
Conceptual overview of tidal trapping scenarios in a dead-end side channel. The top view shows the cross-sectionally averaged salinity, while the cross-sectional plots below further illustrate the assumed exchange mechanisms. **a** Pure advective out-of-phase exchange results in a salt anomaly that causes an up-estuary salt flux due to differences in velocity phasing between flow in the main channel and in the trap. **b** Out-of-phase exchange with complete internal mixing: Similar to **a**, but the trapped salinity field exits the trap fully mixed. **c** Pure diffusive exchange: Continuous diffusive exchange causes the development of salt anomalies in the main channel and an up-estuary salt flux. The diffusive exchange is visualized here as a vertically sheared exchange flow in the cross-section plots, though the diffusive exchange flow could also result from other mechanisms. **d** Out-of-phase and diffusive exchange: This situation involves channel-trap exchange driven by both types of exchange mechanisms

#### Formulations for Channel-Trap Exchange

We describe different types of channel-trap exchange by using different forms for the local source-sink term *I*(*x*) in Eq. 4. The types of exchange are illustrated in Fig. 2. The first two cases test theoretical forms for advective out-of-phase exchange as studied by Dronkers (1978) and MacVean and Stacey (2011). The third case evaluates pure diffusive exchange (Okubo, 1973), while the final case examines the combined effect of advective out-of-phase and diffusive exchange.

**Case 1: Pure advective out-of-phase channel-trap exchange**

In this scenario, salt is extracted from the main channel by the trap at a rate determined by the discharge amplitude $\hat{q}_t = B_t \eta _0\omega$, starting from $t=0$, which is chosen to coincide with slack water in the trap. After the flow reversal in the trap at $t = T/2$, the trapped salinity field is advected back to the main channel, creating a mirrored image of the flood salinity signal:

8 $$\begin{aligned} I(x) = \left\{ \begin{array}{ll} \hat{q}_t \sin (\omega t)\cdot s(x,t) & 0 \le t \le T/2; \\ \hat{q}_t \sin (\omega t) \cdot s(x,T-t) & T/2 \le t \le T, \end{array}\right.  \end{aligned}$$

with *s*(*x*, *t*) the salinity in the main channel. This case is most realistic for shallow systems with high cross-sectional average currents, where diffusive exchange over the trap entrance is relatively small and stratification within the trap does not occur (see Fig. 2a).

**Table 1:** Overview of the assumed geometry and forcing in the channel and trap for the simulations performed in the indicated sections

Section	Case	$\frac{B_tH_t}{A_c}$(-)	$\frac{\Delta x}{L_t}$(-)	$K_b$(m$^2$/s)	$u_r$ (m/s)	$\hat{U}_{c,d}$(m/s)	$\alpha _{d}$ (rad)	$\eta _0$(m)	$K_t$(m$^2$/s)
“Changes in the Along-Channel Salinity Profile (Case 1)”	1	24	0.04	20	0.004	1	$\frac{1}{4}\pi$	1	−
“Results for Different Types of Channel-Trap Exchange (Cases 1–4)”	1	24	0.04	20	0.004	1	0$\cdots$ $\frac{1}{2}\pi$	1	−
	2	24	0.04	20	0.004	1	0$\cdots$ $\frac{1}{2}\pi$	1	−
	3	24	0.04	20	0.004	1	0$\cdots$ $\frac{1}{2}\pi$	1	180
	4	24	0.04	20	0.004	1	0$\cdots$ $\frac{1}{2}\pi$	1	180
“Transitions Between Advective Out-of-Phase and Diffusion Dominated Channel-Trap Exchange (Case 4)”	4	24	0.04	20	0.004	1	0$\cdots$ $\frac{1}{2}\pi$	0.5$\cdots$2	1$\cdots$3200
“Influence of Background Dispersion in the Main Channel on the Effect of Channel-Trap Exchange”	1	24	0.04	20, 2000	0.001, 0.1	1	0$\cdots$ $\frac{1}{2}\pi$	1	−

**Case 2: Advective out-of-phase channel-trap exchange with mixing**

For the second case, it is assumed that the salinity field, which enters the trap during the flood phase, is fully mixed in the trap before it exits the trap (see Fig. 2b):

9 $$\begin{aligned} I(x) = \left\{ \begin{array}{ll} \hat{q}_t \sin (\omega t)\cdot s(x,t) & 0 \le t \le T/2; \\ \hat{q}_t \sin (\omega t)\cdot s_{ebb} & T/2 \le t \le T, \end{array}\right.  \end{aligned}$$

and the salinity during ebb is given by $s_{ebb} = \frac{2}{T}\int _0^{T/2} s(x,t) dt$. This formulation mimics the effect of mixing within the trap of the salinity field that enters during the flood. It is most realistic for shallow systems where the currents in the trap are substantially reduced, allowing for internal mixing (Garcia et al., 2022).

**Case 3: Pure diffusive channel-trap exchange**

Case 3 investigates pure diffusive exchange (Fig. 2c). A numerical sub-domain simulates the trap’s salinity by solving the diffusion equation with the salinity in the main channel serving as a time-varying boundary condition. The diffusive salt flux across this boundary is determined by a lateral diffusion coefficient, $K_t$, specified for the trap, and the salt flux across the boundary serves as the source-sink term, *I*(*x*). For the initial salinity field, the first tidal cycle is simulated repeatedly to spin up the salinity in the trap until the maximum change over one tidal cycle is less than 0.05 psu. This scenario is particularly relevant for systems where the depth-averaged current velocity in the trap is weak, and the exchange is predominantly governed by diffusive mechanisms. For example, this is generally the case for harbors or channel irregularities, where the depth-averaged current in the trap is weak due to their depth and limited length.

**Case 4: advective out-of-phase and diffusive channel-trap exchange**

In case 4, the combined effect of out-of-phase and diffusive exchange is explored (see Fig. 2d). Similar to case 3, a numerical sub-domain is set up to simulate the salinity dynamics within the trap, but with a mean current. An adjusted version of Eq. 4 is used to account for the water level variations. The same spin-up procedure as described for case 3 is used to obtain an initial salinity field.

#### Performed Model Simulations

To investigate the influence of different types of channel-trap exchange and key estuarine parameters on the dispersive effect within the main channel, we performed a systematic set of model simulations to demonstrate the effect of channel-trap exchange (Table 1).

In all simulations, a model domain of 200 km was discretized with variable cell sizes: 250 m near the mouth, 125 m around the trap, and 500 m in the upper estuary. A tidal period *T* of 12 h is assumed, and a time step of 100 s was used for time discretization. Furthermore, a system with a sufficiently long intrusion length is simulated to ensure the trap is not influenced by boundaries, and a long dispersive adjustment time is desired to limit the influence of background dispersion ($T_D$ $\gg$ *T*). To accomplish this, a cross-sectional area $A_c = 2500$ m$^2$ and a river velocity $u_r = -0.004$ m/s and background diffusion of $K_b = 20$ m$^2$/s are used to reach a salt intrusion length $L_s$ (1 psu isohaline) of approximately 47 km. In practice, typical values $K_b$ range from 100 to 300 m$^2$/s (Fischer et al., 1979), although reported values vary considerably, ranging from approximately 20 to 2000 m$^2$/s (Fischer et al., 1979; Savenije, 2006; Kuijper & Van Rijn, 2011). We use the lower limit of $K_b$ in this study. To initialize the model with a true equilibrium salinity distribution, the model without any source/sink terms was run until a new equilibrium was reached (see Fig. 1), which was then used to initialize the model simulations exploring the channel-trap exchange. In these simulations, a trap is located at $x_h=40$ km with $x_h$ indicating the center location of the trap in the main channel. The trap has an entrance width $\Delta x = 250$ m, a basin length $B_t=12000$ m, and a depth $H_t=5$ m. There is no tidally averaged net discharge through the trap, and therefore, it can be considered a dead-end side channel. A reference simulation is performed without the trap for comparison.

Table 1 gives an overview of the simulations of this study. Firstly, we simulate a purely advective out-of-phase exchange between channel and trap and report on the changes in the salinity in the main channel in Section “Changes in the Along-Channel Salinity Profile (Case 1)”. Simulations are performed using different formulations for the channel-trap exchange, while varying the velocity phase for the current in the down-estuary reach of the main channel. For simulations reported in Section “Results for Different Types of Channel-Trap Exchange (Cases 1–4)”, $\alpha _d$ is varied between 0 and $\frac{\pi }{2}$, such that the velocity phase difference between the downstream flow and the flow in the trap is varied between the smallest and largest physically realistic values. The simulations reported in Section “Transitions Between Advective Out-of-Phase and Diffusion Dominated Channel-Trap Exchange (Case 4)” aim to explore the transition from advective out-of-phase to diffusive channel-trap exchange (case 4). For these simulations, the strength of the lateral diffusion coefficient is varied between 1 and 3200 m$^2$/s, and the current in the trap is varied by changing the tidal amplitude $\eta _0$ between 0.5 and 2 m. Finally, as discussed in Section “Model Set-up”, different combinations for $u_r$ and $K_b$ may yield the same salt curve, while they vary in their diffusive and advective time-scales. Altering the diffusive time-scale influences the additional salt flux. To illustrate this relationship, in Section “Influence of Background Dispersion in the Main Channel on the Effect of Channel-Trap Exchange”, experiments are conducted assuming pure advective out-of-phase dynamics, in which $K_b$ is set equal to 20 and 2000 m$^2$/s, with river-induced currents of 0.1 and 0.001 m/s (Table 1). To allow for a meaningful comparison, both sets of experiments are initialized with the salt curve obtained from Eq. 5.

### Analysis of Model Results

#### Decomposition of the Tidally Averaged Salt Fluxes

To differentiate between the various exchange mechanisms in the model and quantify the additional (tidal) salt flux introduced by the trap, the tidally averaged salt flux *F* through a cross-section in the main channel is decomposed. Both the flow and the salinity signal are decomposed into a subtidal mean and tidally varying component indicated with the subscripts $$_0$$ and $$_1$$, respectively. This decomposition is used to quantify the flushing by the river discharge flow ($$F_0 = Q_rs_0 $$) and the opposing salt flux related to tidal dispersion ($$F_1 = A_c\langle u_1s_1 \rangle $$), with the operator $$\langle \rangle $$ indicating the tidal average. In the cross-sectional averaged model, the net salt flux through a cross-section is given by

10 $$\begin{aligned} F = A_c\biggl ( u_0s_0 + \langle u_1s_1 \rangle + K_b \frac{\partial s_0}{\partial x} \biggr ), \end{aligned}$$

where the third term represents the parameterized salt flux due to background dispersion ($F_b = A_cK_b\frac{\partial s_0}{\partial x}$). The system is in (dynamic) equilibrium if the net salt flux through the cross-section equals zero throughout the entire model domain and $F_0$ is balanced by $F_1$ and $F_b$. Using a Fickian diffusion coefficients, the up-estuary salt fluxes can be expressed into an effective dispersion coefficient $K_\text {eff} = F_{1} /\bigl (A_c \frac{\partial s_0}{\partial x}\bigr ) + K_b$. This effective dispersion coefficient captures all modelled dispersion mechanisms and thus does not only represent the effect of tidal trapping.

#### Quantification of the Trap-Induced Additional Salt Fluxes

To quantify the additional tidally averaged salt flux (directly) resulting from channel-trap exchange, two models are run for one tidal cycle: one with a trap and the other without. Both simulations start from the same initial salinity field. The increase in the tidally averaged salt flux through a cross-section near the trap in the simulation with the trap, compared to the reference simulation, is defined as the additional salt flux caused by the trap, $\tilde{F}_\text {trp}(x)$. This additional salt flux is used to quantify the effect of a trap under different estuarine conditions.

**Fig. 3:**
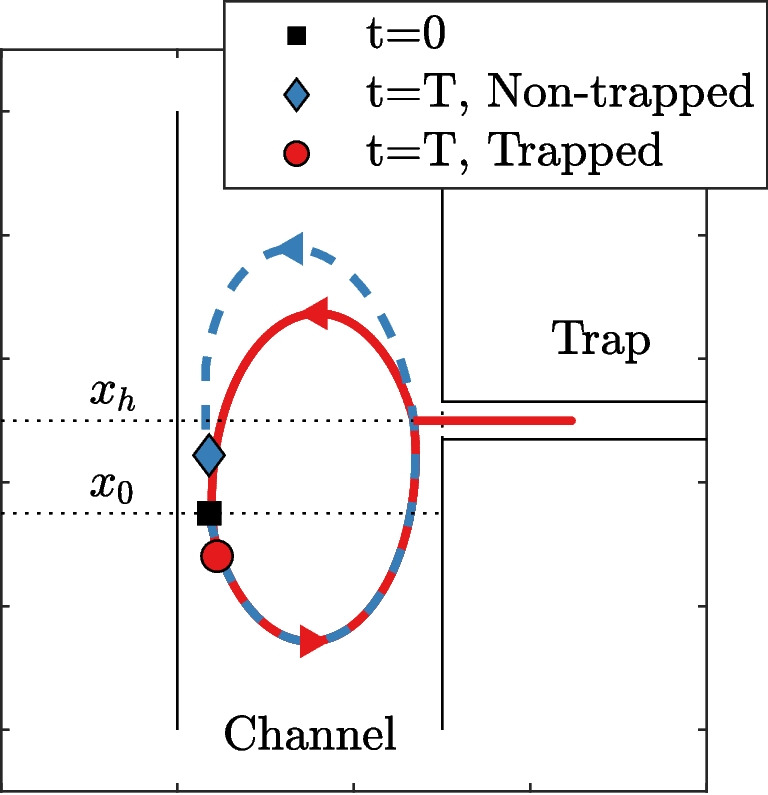
Illustration of the Lagrangian trajectory of two water parcels in a scenario where the velocity in the trap and the main channel are out-of-phase. One volume gets trapped, experiencing a displacement in the down-estuary direction, while the other remains in the main channel and is displaced in the up-estuary direction. Note that this Lagrangian displacement differs from the net up-estuary displacement $\delta$ calculated for a cross-section. The dimensions of the channel are not drawn to scale for clarity. This situation corresponds to Fig. 4

Analytically reproducing this additional salt flux from the observed channel-trap exchange is not straightforward. Here, we use an adjusted version of the balance proposed by Dronkers (1978) (see Eq. 20 in Dronkers (1978)) to estimate the trapping effect over the excursion length, $F_\text {trp}(x)$. Here, we distinguish between the effects of advective out-of-phase exchange and diffusive channel–trap exchange by decomposing the instantaneous salt flux over the trap entrance *f* (where a positive value indicates a flux into the trap), into two component the cross-sectionally averaged advective salt flux $f_{A}$, and the diffusive salt flux $f_{D}$, parameterized by the lateral diffusion coefficient $K_t$. If the velocity in the main channel and trap is in phase, the additional tide-averaged salt flux through a cross-section at $x=x_0$ is equal to the net salt flux over the trap entrance during the time interval between $t_{0,f}$ and $t_{0,e}$. Those instants correspond to the moments when particles, departing from $x_0$ at $t=0$ in the main channel, pass by the trap during flood and ebb, respectively. If there is a velocity phase difference, a net salt flux is generated by the relative motion between the channel and the trap. A trapped parcel experiences a down-estuary displacement from its initial location at $t=0$, whereas a parcel remaining in the channel experiences an up-estuary displacement. This is illustrated in Fig. 3. The balance for the additional tide-averaged salt flux through a cross-section $\tilde{F}_\text {trp}(x)$ is given by

11 $$\begin{aligned} \tilde{F}_\text {trp}(x_0) = {\left\{ \begin{array}{ll} \underbrace{ \int _{t_{0,f}} ^{t_{0,e}}f_{D}dt }_{\tilde{F}_{D}}+\underbrace{\int _{t_{0,f}}^{t_{0,e}}f_{A}dt }_{\tilde{F}_{A}}+\underbrace{\int _{-\delta }^0 s(x_0,0)A_c dx }_{\tilde{F}_{C}}, & x_1\le x_0< x_h \\ \underbrace{ \int _{t_{0,f}}^{t_{0,e}}-f_{D}dt }_{\tilde{F}_{D}}+\underbrace{\int _{t_{0,f}}^{t_{0,e}}-f_{A}dt }_{\tilde{F}_{A}}+\underbrace{\int _{-\delta }^0 s(x_0,0) A_c dx }_{\tilde{F}_{C}}, & x_h < x_0 \le x_2 \end{array}\right. } \end{aligned}$$

where $x_1 = x_h-L_t+\frac{u_c}{\omega } (1-\cos (\alpha _d))$ and $x_2 = x_h+\frac{u_c}{\omega } (1-\cos (\alpha _d))$ are the up- and down-estuary limits of the additional salt flux. The tilde $\tilde{\phantom{0}}$ is used to indicate that this is an analytical estimate of the additional salt flux. The first term ($\tilde{F}_D$) represents the net additional salt flux from diffusive exchange with the trap. The second term ($\tilde{F}_{A}$) represents the net salt flux due to advective exchange with the trap, which is typically negative when considering pure out-of-phase exchange. This negative flux results from the fact that, by definition, a larger volume leaves the trap during the evaluated period due to the effect of the relative motion between the channel and the trap. The third term ($\tilde{F}_C$) represents the salt flux resulting from the need to compensate for the net volume exiting the trap (volume conservation). This is achieved by a net displacement $\delta$ in the up-estuary direction. This displacement is calculated from the net discharge from the trap during the evaluated period: $\delta (x) =\int _{t_{0,f}} ^{t_{0,e}}\Delta x \hat{q}_t \sin (\omega t) dt / A_c$, where the integral is analogous to the second term ($\tilde{F}_A$) but for the exchange of volume. Hence, if diffusive exchange is negligible, the balance between the down-estuary directed term $\tilde{F}_{A}$ and the compensating term $\tilde{F}_{C}$ describes the effect of pure advective out-of-phase exchange.

The ability of Eq. 11 to reproduce the additional tidally averaged salt flux and quantify the contribution of the different exchange mechanisms is demonstrated in Section “Results”.

### Limitations

The idealized one-dimensional (1D) model adopted to explore tidal trapping has limitations. Many physical processes are either greatly simplified, represented by a single (dispersion) coefficient, or not accounted for at all. For example, the model does not capture stratification that may be generated due to the reduction in current velocity within the trap (Garcia & Geyer, 2022). Additionally, the influence of lateral salinity variations in the main channel, which depend on the geometry of the system (Schulz et al., 2015), on the trapping process is neglected. Exchange at estuarine junctions is often accompanied by frontal formation (Corlett & Geyer, 2020), which can significantly influence both stratification and mixing (Giddings et al., 2012; Bo & Ralston, 2022). These types of processes and interactions remain unexplored. This notwithstanding, the essential mechanism of out-of-phase salinity exchange referred to as tidal trapping is captured in our 1D modelling framework.

**Fig. 4:**
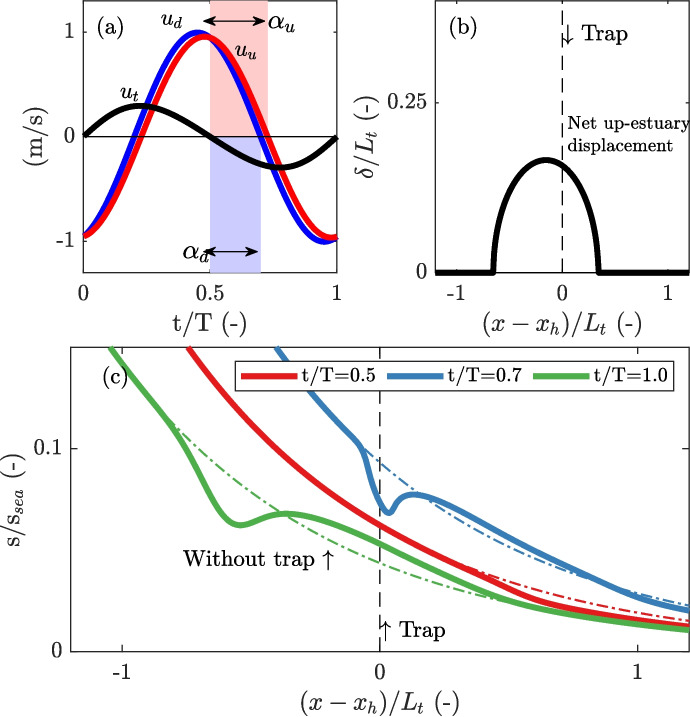
Illustration of the modelled velocities alongside intertidal salinity levels, demonstrating the development of salt anomalies driven by the net displacement of trapped and non-trapped particles, assuming pure advective out-of-phase exchange. **a** Illustration of the current velocities and resulting phase differences. The current velocity up-estuary of the trap, $u_{d}$, results from the assumed current velocity profile in the reference run and the velocity phase difference between the down-estuary section of the main channel and the trap. **b** Trap-induced net up−estuary displacement $\delta$ over a tidal cycle, shown as a function of the initial location at $t/T = 0$ relative to the trap, normalized by the excursion length. **c** Simulated salinity in the reference model (dashed lines) and in the simulation with source-sink terms for out-of-phase exchange (solid lines) at various moments in time, both at and after the flow reversal in the trap (occurring at $t/T=0.5$). In **b** and **c**, the x-axis shows that the distance in the main channel from the trap $(x - x_h)$ is normalized by the tidal excursion $L_t$

## Results

### Changes in the Along-Channel Salinity Profile (Case 1)

This section examines how salinity along the main channel changes under the influence of pure advective out-of-phase exchange with a trap (case 1) and quantifies the associated additional salt flux.

**Fig. 5:**
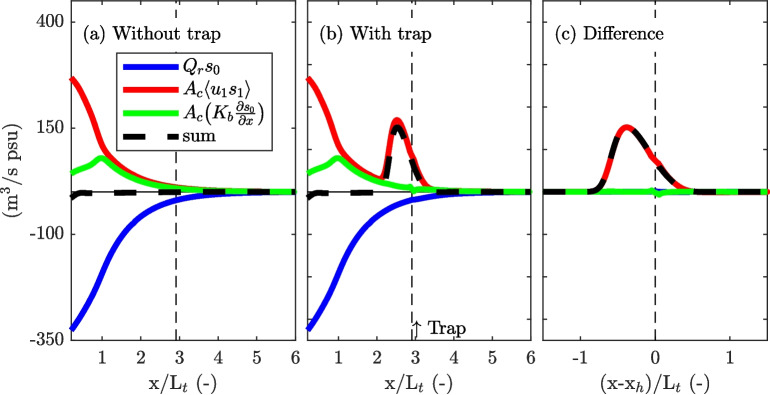
Example of the salt fluxes in the simulation with and without a trap corresponding to the situation depicted in Fig. 4. **a** Along-channel salt fluxes for the reference simulation (no trap). The along-channel coordinate is normalized by the tidal excursion length. **b** Same as **a**, but for the simulation with the trap, modelled using the source-sink terms. **c** Difference between the two simulations after one tidal cycle

Figure 4 presents the simulated current velocities, the calculated net displacement in up-estuary direction, and the resulting salt anomaly from the channel-trap exchange, which develops after a full tidal cycle. The adopted settings result in an up-estuary velocity amplitude $\hat{U}_{c,u} \approx 0.95$ m/s with a velocity phase $\alpha _u \approx 0.29 \pi$ (Fig. 4a). A greater velocity phase (i.e. later onsets of flood and ebb) in the up-estuary reach of the main channel causes non-trapped parcels to experience an up-estuary displacement from their initial position. In contrast, a smaller velocity phase in the trap (i.e. earlier onsets of flood and ebb) causes trapped particles to return earlier to the main channel and experience a down-estuary displacement (Fig. 3). Figure 4b shows the calculated net up-estuary displacement associated with an up-estuary volume flux required to balance the down-estuary volume flux induced by the relative motion between the channel and the trap at each cross-section of the main channel. Figure 4c shows the development of a negative salt anomaly that develops in the main channel when the flow in the trap reverses ($t/T > 0.5$). During the period that the trap is in flood phase, saline water is only extracted by the trap, and the trap does not generate a gradient in the along-channel salinity field. Differences in the salinity field between the two simulations at $t/T = 0.5$ are solely caused by along-channel gradient in the along-channel velocity. However, when the flow in the trap reverses, a negative salt anomaly develops in the main channel at $x_h$, caused by the re-discharge of relatively lower saline water. This negative anomaly develops because the re-discharged water re-enters the main channel down-estuary from its initial location in the tidal excursion. The non-trapped parcels experience an up-estuary displacement due to the greater velocity phase and create the observed positive salt anomaly upstream of the trap. After one tidal cycle ($t/T = 1.0$), the channel-trap exchange has resulted in a redistribution of salt over the tidal excursion length.

The effect of the developed salt anomaly on the tidally averaged salt fluxes is shown in Fig. 5. Note that both simulations start from the equilibrium salinity distribution obtained for the situation without a trap. In both simulations, the river discharge introduces a phase shift that is smaller than in the case of quadrature, which results in a tidal salt flux over the salt intrusion length (Dijkstra et al., 2022). Furthermore, an increase in the tidal salt flux is introduced by the seaward boundary conditions and is visible near the mouth ($0 \le x \le L_t$), resulting from a difference in salinity during flood and ebb similar to jet-sink exchange (Stommel & Former, 1952). In this region, the salt flux due to background dispersion is reduced, owing to a weaker subtidal salinity gradient (also see Fig. 1a). However, in the system with a trap, an additional net salt flux is visible near the trap. Relative to the reference run, the decomposed salt fluxes are equal in both simulations, except for the additional tidal salt flux seen in the proximity of the trap, which is introduced by the channel-trap exchange illustrated in Fig. 4.

### Results for Different Types of Channel-Trap Exchange (Cases 1–4)

Next, this section studies how salinity anomalies and salt fluxes in the channel differ for alternative types of channel-trap exchange (see Section “Formulations for Channel-Trap Exchange”). We use settings as tabulated in Table 1.

**Fig. 6:**
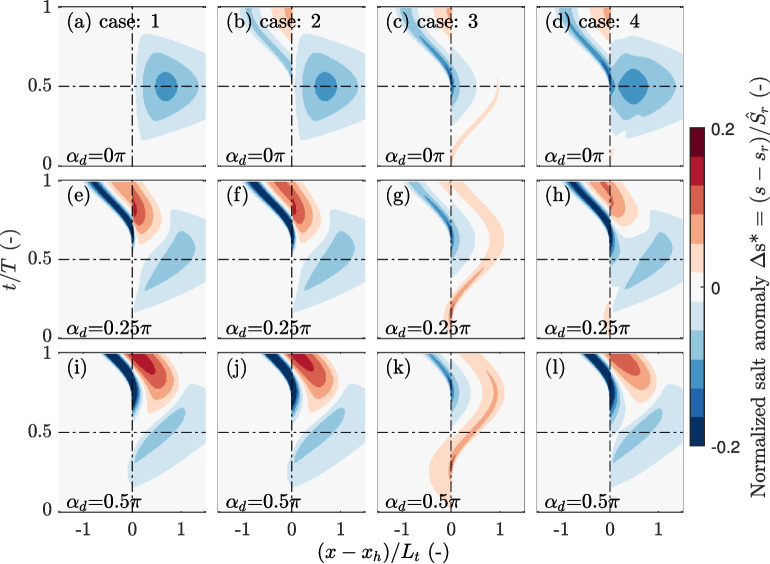
Spatio-temporal development of the normalized salt anomaly $\Delta s^*$ in the main channel resulting from channel-trap exchange. Cases 1 through 4 (i.e. definition of the source-sink term) correspond to the columns, and rows differ in the assumed velocity phase differences $\alpha _d$. The normalized salt anomaly $\Delta s^*$ is defined as the difference in the salinity in the main runs compared to the reference run normalized by the salinity amplitude: $\Delta s^* = (s-s_r)/\hat{S}_r$, with $s_r$ the salinity in the reference simulation and $\hat{S_r}$ denoting the undisturbed salinity amplitude in the reference run. For the x-axis, the distance in the main channel from the trap $(x - x_h)$ is normalized by the tidal excursion $L_t$

Figure 6 presents the spatio-temporal development of the salt anomaly simulated for the different cases and assumed values for $\alpha _d$. The salt anomaly is defined as the difference with the reference run, $\Delta s= s-s_r$. When assuming pure advective out-of-phase exchange (case 1), the resulting salt anomaly clearly increases with $\alpha _d$ and vanishes for $\alpha _d = 0$. In the latter case, the observed intertidal differences are solely the result of reduced flow velocities upstream of the trap. However, when the salt transported into the trap is assumed to be mixed before it flows back into the channel (case 2), a salt anomaly develops also in the absence of a phase difference (Fig. 6a, b). Mixing in the trap causes the salinity of the rejoining water masses to differ from the water mass remaining in the main channel (Dronkers, 1978; Garcia et al., 2022). However, the influence of mixing in the trap on the developed salt anomaly is less evident for higher velocity phase differences compared to pure advective out-of-phase exchange (Fig. 6i,j). The salt anomaly that is observed for pure diffusive exchange (case 3) develops in a distinctive manner. The channel-trap exchange is driven by a lateral salinity gradient, which is set up by the tidal motion in the main channel. This results in a diffusive salt flux from the trap that is in quadrature with the main channel velocity. A negative salt anomaly develops between the periods when the main channel current reaches peak flood and ebb (with $\alpha _d = 0$, this is between $\frac{t}{T }= \frac{1}{4}$ and $\frac{t}{T }= \frac{3}{4}$), causing the re-discharge of relatively low saline water from the trap (Fig. 6c). For the scenario with both advective out-of-phase and diffusive exchange (case 4), the results are similar to the scenario for advective out-of-phase exchange with mixing in the trap (case 2), but driven by distinct processes.

**Fig. 7:**
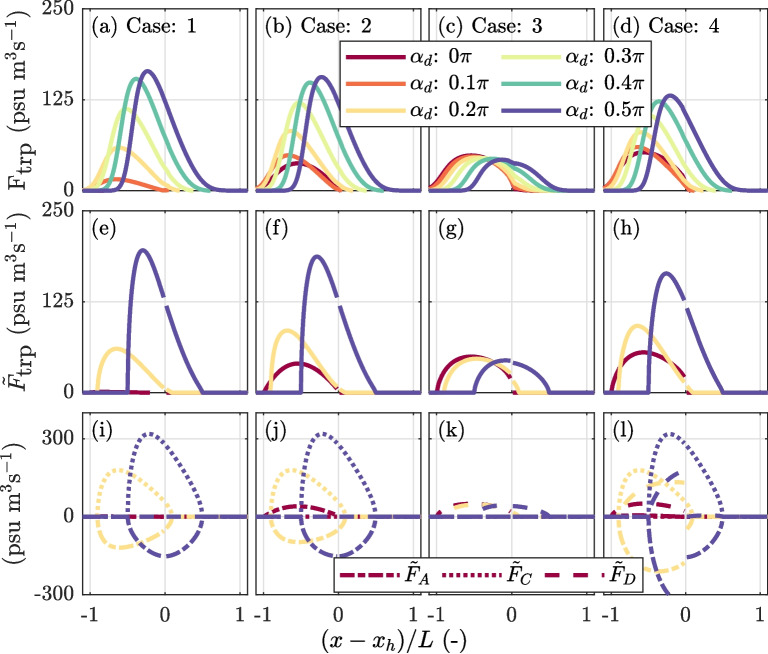
Additional salt flux in the main channel and its contributors resulting from channel-trap exchange for alternative channel-trap exchange mechanisms and assumed velocity phase differences ($\alpha _d$). **a**–**d** The additional salt flux is determined by calculating the difference in salt fluxes between the simulations with and without the trap. **e**–**h** Additional salt fluxes reproduced based on Eq. 11. **i**–**l** Contribution of the individual terms of Eq. 11 that quantify the salt flux resulting from advective exchange over the trap entrance $\tilde{F}_{A}$, the salt flux resulting from the up-estuary net displacement $\tilde{F}_{c}$ (required to balance the relative motion between channel and trap), and the salt flux resulting from the continuous diffusive channel-trap exchange, $\tilde{F}_{D}$. At the x-axis, the distance in the main channel from the trap $(x - x_h)$ is normalized by the tidal excursion $L_t$

Figure 7 presents the additional salt flux from the salt flux decomposition, the reproduced additional salt flux calculated using Eq. 11, and the contributions of the individual terms for the different cases. For pure advective out-of-phase exchange (case 1), it is evident that the additional salt flux increases with the velocity phase difference (Fig. 7a). This additional salt flux is fairly well reproduced through Eq. 11 (Fig. 7e). The observed differences between the calculated salt flux and the salt flux obtained from the flux decomposition are attributed to the fact that in the calculated salt flux, diffusion in the main channel is ignored. The reproduced salt fluxes emphasize the influence of relative displacement and show that the additional salt flux is a balance between the down-estuary directed flux from the trap $\tilde{F}_{A}$ and the compensating salt flux resulting from the net up-estuary displacement $\tilde{F}_{C}$ (Fig. 7i). Generally, the location where the additional salt flux is largest is near the peak in the net displacement, $\delta$, but it also depends on the shape of the along-channel salinity distribution. In our case, due to the exponentially decaying salinity field, the additional salt flux is slightly skewed, with the peak located down-estuary from the peak in displacement.

The effect of mixing in the trap (case 2) when $\alpha _d = 0$ results in a positive contribution of $\tilde{F}_{A}$ (Fig. 7j). The influence reduces at higher velocity phase differences due to the structure of the imported salinity field. Furthermore, it is shown that the additional salt flux directly caused by the channel-trap exchange is not necessarily maximum at the location of the trap where the salt anomaly enters the channel (Garcia et al., 2022). Figure 7b shows that the additional salt flux is close to zero at the junction and peaks at $\frac{1}{2}L_t$ when $\alpha _d$ is small.

For the diffusive trap (case 3), the additional salt flux is solely determined by $\tilde{F}_D$ (Fig. 7c, g, k). Theoretically, in that case, the additional salt flux is independent of $\alpha _d$. But in the performed experiments, $\tilde{F}_D$ is slightly influenced by the initial salinity field in the trap. The salt fluxes resulting from both advective out-of-phase and diffusive exchange (case 4) show a similar development with an increase in $\alpha _d$ compared to the case where mixing in the trap is assumed (Fig. 7d). For small velocity phase differences, a net salt flux is generated through diffusive exchange, quantified by $\tilde{F}_D$. However, when the velocity phase difference is large, the resulting additional salt fluxes are lower compared to cases 1 and 2 (Fig. 7d). For higher velocity phase differences, the reduction in the additional salt flux is caused by an increase in the magnitude of $\tilde{F}_{A}$, which is not compensated by $\tilde{F}_D$. The importance of advective out-of-phase versus diffusive exchange is further explored in the next section.

**Fig. 8:**
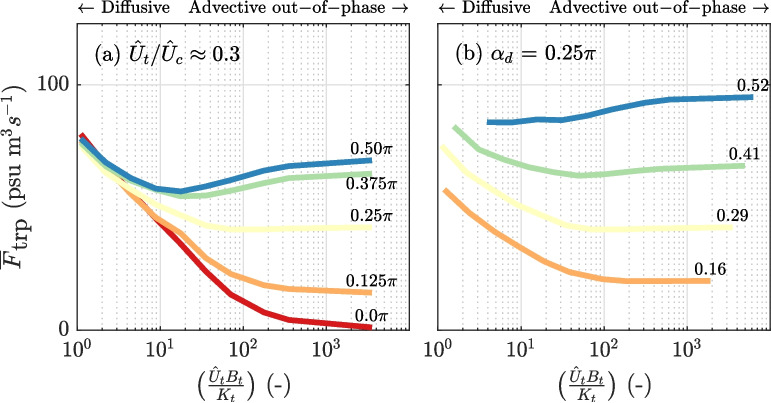
Overview of the tidal excursion-averaged additional salt flux, $\overline{F}_\text {trp}$, as a function of Péclet number, demonstrating the transition from diffusive to out-of-phase dominated exchange. The Péclet number is defined as $P_e =\frac{\hat{U}_tB_t}{ K_t}$, with $\hat{U}_t$ the velocity amplitude in the trap, $B_t$ is the length of the trap, and $K_t$ is the assumed lateral diffusion coefficient. **a** Tidal excursion-averaged additional salt flux for alternative velocity phase differences $\alpha _d$, with $\hat{U}_t/\hat{U}_c = 0.3$. **b** Same as **a**, but with $\alpha _d = 0.25\pi$ and varying $\hat{U}_t/\hat{U}_c$

### Transitions Between Advective Out-of-Phase and Diffusion Dominated Channel-Trap Exchange (Case 4)

For case 4, the channel-trap exchange occurs through a combination of advective out-of-phase and diffusive exchange. To differentiate between advective out-of-phase and diffusion−dominated exchange, we characterize the exchange using the Péclet number. The length of the basin $B_t$ serves as the typical length scale. The advective time-scale $T_A$ is determined from the trap’s flushing time, given by $T_A= B_t/u_t$. For the diffusive time-scale, an effective migration rate within the trap is assumed, equal to $u_{f} = K_t/B_t$, resulting in $T_D = B_t^2/K_t$. The ratio of the two time-scales yields a Péclet number $P_e = \frac{T_D}{T_A} =\frac{\hat{U}_tB_t}{ K_t}$. MacVean and Stacey (2011) used a scaled Péclet number, assuming that the characteristic time-scales of advective and diffusive exchange differ. However, both processes operate over a typical time-scale equal to *T* when the trap length $B_t$ is sufficiently long. Therefore, here we consider the characteristic time-scale required to flush the trap through advective and diffusive transport. Furthermore, a tidal excursion-averaged additional salt flux, $\overline{F}_\text {trp}$, is calculated to quantify the trap’s effect:

12 $$\begin{aligned} \overline{F}_\text {trp} = \frac{1}{L_t} \int _{x_1} ^{x_2}F_\text {trp} dx \end{aligned}$$

where the overbar $\bar{\phantom{0}}$ is used to indicate a tidal excursion-averaged property.

Figure 8 presents the tidal excursion−averaged dispersion coefficient across the tested Péclet numbers. The results indicate that the additional salt flux, $\overline{F}_\text {trp}$, does not necessarily increase when transitioning from out-of-phase to diffusive dominated channel-trap exchange (i.e. from right to left in Fig. 8a). Instead, it depends on velocity phase difference $\alpha _d$. An increase in the relative importance of diffusive transport enhances the additional dispersion coefficient when $\alpha _d$ is small (e.g. red line in Fig. 8a). However, for large values of $\alpha _d$, where advective out-of-phase exchange significantly contributes to the additional salt flux, increasing the diffusive transport tends to diminish the trap effect, as noted in Section “Results for Different Types of Channel-Trap Exchange (Cases 1–4)”. This is more pronounced when the normalized current velocity in the trap, $\hat{U}_t/\hat{U}_c$, is high (Fig. 8b). At low Péclet numbers, the resulting tidal excursion−averaged dispersion coefficient behaves independently of tidal characteristics and is essentially determined by the magnitude of $K_t$ and the trap length $B_t$. The effectiveness of the trap diminishes at low Péclet numbers when the diffusive salt front can reach the end within half a tidal cycle, leading to reduced residence times as a consequence. So, in conclusion, the presence of a diffusive salt flux can diminish the effect of the advective out-of-phase exchange on the salt fluxes in the channel.

**Fig. 9:**
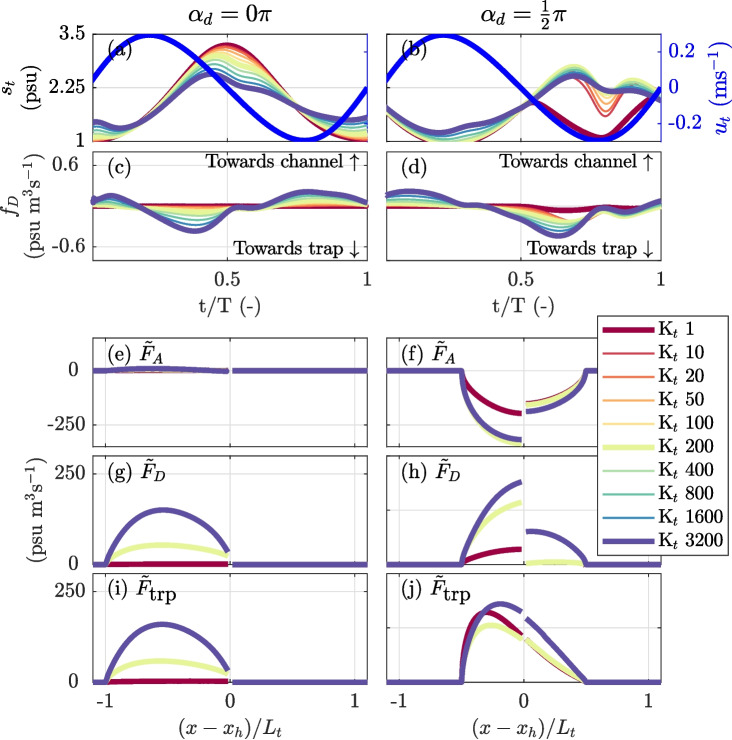
Salt fluxes at the trap entrance and resulting additional salt flux in the main channel for varying lateral diffusion coefficients, $K_t$, illustrating the transition from out-of-phase toward diffusive dominated channel-trap exchange. This is shown for two contrasting scenarios: (1) no velocity phase difference ($\alpha _d = 0 \pi$) and (2) the largest physically realistic phase difference ($\alpha _d = 0.5 \pi$). In both scenarios, $\hat{U}_t/\hat{U}_c = 0.30$ m/s. **a** and **b** Salinity and current near the trap entrance. **c** and **d** Instantaneous diffusive salt flux $f_D$ at the trap entrance. **e** and **f** Predicted additional salt flux in the main channel resulting from cross-sectional average transport at the trap entrance, $\tilde{F}_{A}$. **g** and **h** Same as panel **e** and **f** but for diffusive exchange, $\tilde{F}_{D}$. **g** and **h** Predicted total additional salt flux resulting from channel-trap exchange, $\tilde{F}_\text {trp}$

To explain why the presence of a diffusive salt flux can diminish the effect of the advective out-of-phase exchange, we examine the exchange over the trap entrance for two contrasting scenarios: no phase difference ($\alpha _d = 0\pi$) and the largest realistic phase difference ($\alpha _d = 0.5\pi$), as shown in Fig. 9. Focussing on the instantaneous diffusive salt flux $f_D$ (Fig. 9c, d), it is evident that for $\alpha _d = 0.5\pi$, the diffusive exchange shows an asymmetry in the magnitude and duration for which $f_D$ is directed toward the trap, even for lower $K_t$ values, which contrasts with $\alpha _d = 0\pi$. The prolonged period during which the diffusive salt flux is directed toward the trap and increases in magnitude results from the development of a strong lateral salinity gradient near the trap entrance as the trap starts to empty. Due to the velocity phase difference, the salinity in the main channel increases, while near the trap entrance, the salinity decreases as the trap empties, generating a sharp salinity gradient that promotes a diffusive flux into the trap (phase-induced diffusive salt flux). This diffusive flux increases the salinity near the trap entrance compared to the case of nearly pure advective out-of-phase exchange (Fig. 9b). In contrast, with $\alpha _d = 0\pi$, an increase in $K_t$ causes continuous diffusive exchange between the channel and the trap, resulting in a nearly symmetrical diffusive salt flux $f_D$ for both flood and ebb periods. A sharp lateral salinity gradient near the trap entrance does not develop because the salinity of the rejoining water masses is nearly the same.

Panels 9e-j illustrate the additional salt fluxes predicted by Eq. 11, aiming to clarify the role of phase-induced diffusive salt fluxes. For $\alpha _d = 0\pi$, the additional salt flux is governed by diffusive exchange, $\tilde{F}_{D}$, which increases with $K_t$ (see Fig. 9g). This increase in $\tilde{F}_{D}$ is also observed for $\alpha _d = 0.5\pi$, but is influenced by the phase-induced diffusive salt flux (panel 9h). The raised salinity near the trap entrance while the trap empties (Fig. 9b) enhances $\tilde{F}_A$ (panel 9f) and reduces the contribution of advective out-of-phase exchange, balancing $\tilde{F}_A$ and $\tilde{F}_C$. The increase in $\tilde{F}_{A}$ exceeds that of $\tilde{F}_D$, explaining why the phase-induced diffusive salt flux reduces the resulting additional salt flux. When $K_t$ increased further, the trap’s contribution increases again due to intensified diffusive exchange $\tilde{F}_{D}$. The magnitude of $\tilde{F}_{A}$ decreases slightly as salinity variations at the trap entrance diminish.

Figure 8b illustrates that the phase-induced diffusive reduction in the dispersive effect of the trap is more pronounced when the velocity in the trap (normalized by the main channel velocity) is relatively strong for the same Péclet number. A small current velocity in the trap leads to a shorter distance over which the salinity field is advected into the trap ($L_t \sim \hat{U}_t/\omega$) producing a stronger gradient, since $\frac{\partial s_{t}}{\partial y} \sim \frac{u}{u_t}{}\frac{\partial s}{\partial x}$. A larger velocity in the trap increases the distance over which the salinity field is advected and reduces lateral gradients. For smaller $\hat{U}_t/\hat{U}_c$, a diffusive salt front propagates more easily into the trap and can exceed the distance over which the salinity field is advected by the tidal motion even with a small $K_t$. Conversely, for larger values of $\hat{U}_t/\hat{U}_c$, the salt front propagates less easily into the trap and has to travel further to exceed the excursion limit. Hence, the structure of the salinity field remains determined by the tidal motion, and a diffusive salt flux develops during ebb, causing the phase-induced diffusive damping.

**Fig. 10:**
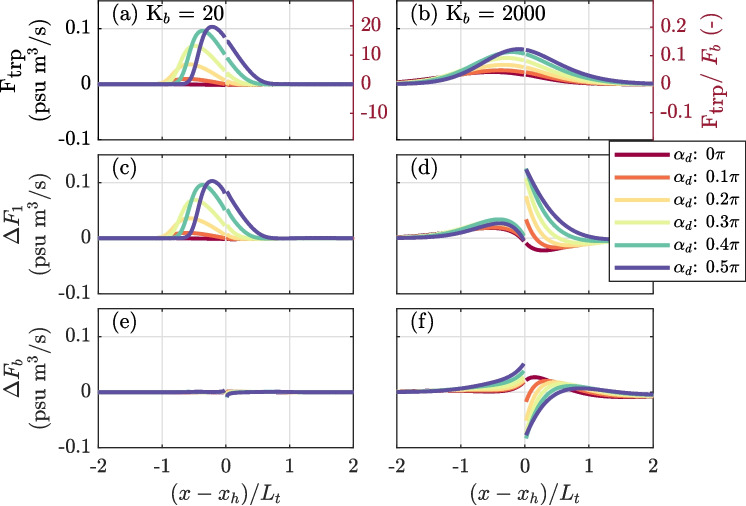
Influence of the background dispersion coefficient, $K_b$, on the salt flux resulting from channel-trap exchange. Shown for two scenarios with a low ($K_b=20$ m$^2$/s) and ($K_b=2000$ m$^2$/s) background diffusion coefficient. **a** and **b** Total additional salt flux, $F_\text {trp}$. **c** and **d** The difference in the tidal contribution, $\Delta F_1$. **e** and **f** The difference in the diffusive contribution $\Delta F_b$

### Influence of Background Dispersion in the Main Channel on the Effect of Channel-Trap Exchange

In previous sections, the additional salt flux was solely attributed to channel-trap exchange. Figure 10 illustrates how the additional salt flux due to trapping is affected by the time-scale of background diffusion in the main channel. With $K_b$ = 20 m$^2$/s, the additional salt flux results fully from an increase in the tidal salt flux, $\Delta F_1 = F_1 - F_{1,r}$, and the extent of the zone in the main channel where the trap effect is visible is approximately equal to $L_t$. In contrast, for $K_b$ = 2000 m$^2$/s, the zone of influence exceeds $L_t$, and the peak additional salt flux is reduced. Furthermore, the additional salt flux is now the result of a balance between tidal and diffusive ($\Delta F_b = F_b - F_{b,r}$) contributions and differs substantially up- and down-estuary of the trap. Even without a velocity phase difference ($\alpha _d = 0$), the trap effect for out-of-phase exchange is not zero. This is explained by the fact that with a higher value for $K_b$, the system responds more rapidly to the salinity gradients that develop during the tidal cycle, influencing the resulting salt fluxes. Furthermore, an increase in the value of $K_b$ implies an increase in the magnitude of $F_b$, which in turn reduces the relative importance of the trap, but also reduces the ability to reproduce $F_\text {trp}$ through Eq. 11.

## Discussion

### Comparison with Existing Theory

In this study, the additional salt flux and associated dispersion coefficients resulting directly from channel-trap exchange were quantified for a single trap, much shorter than the tidal excursion length, across various scenarios. In this section, the additional salt fluxes obtained in Section “Results for Different Types of Channel-Trap Exchange (Cases 1–4)”, expressed as dispersion coefficients, are compared with existing theoretical frameworks derived for different regimes.

**Fig. 11:**
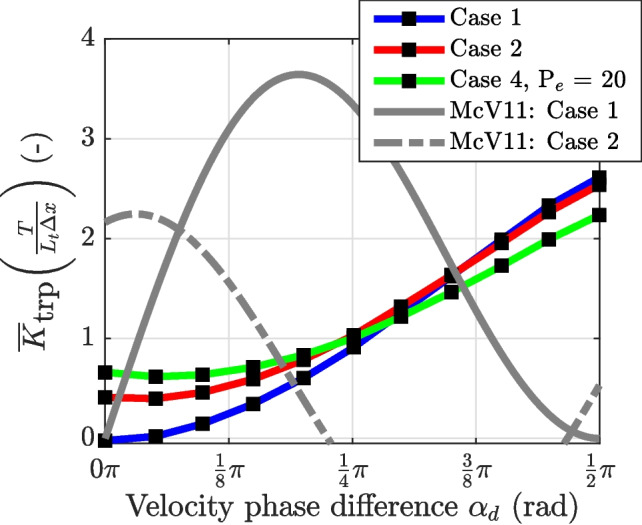
Comparison of the tidal excursion-averaged additional dispersion due to the trap, normalized by $\frac{L_t \Delta x}{T}$, plotted as a function of the phase lead of the trap velocity over the channel velocity (downstream), $\alpha _d$, under different scenarios: pure advective out-of-phase exchange (case 1), advective out-of-phase exchange with mixing in the trap (case 2), and advective out-of-phase exchange with diffusive exchange (case 4, with $P_e = 20$). The numerical results are compared with the analytical framework by MacVean and Stacey (2011), indicated as McV11

#### Pure Advective Out-of-Phase Exchange

Tidal trapping results from advective out-of-phase exchange when diffusive exchange processes are negligible, typically characterized by high Péclet numbers ($P_e> 10^3$), as illustrated in Fig. 8. Figure 11 compares the tidal extent-averaged dispersion coefficient, $\overline{K}_\text {trp}$, with analytical estimates of tidal dispersion for different velocity phase differences. The numerical results indicate that the trap effect increases with the velocity phase difference $\alpha _d$ and scales as $\overline{K}_\text {trp}\sim \sin ^2(\alpha _d)$. This is consistent with the expression by Dronkers (1978) for a continuous trap (see Eq. 3). Note that the analytical framework by MacVean and Stacey (2011) predicts a maximum trapping effect for a channel-trap phase difference of $\frac{1}{4}\pi$, which differs from the present results. This difference is largely explained by the fact that this study accounts for the velocity difference between the upstream and downstream regions relative to the position of the trap. However, this does not fully account for the discrepancy, and the underlying reasons are systematically investigated and discussed in Appendix A.

#### Effect of Mixing in the Trap

Mixing in the trap causes the salinity field leaving the trap to be (partially) mixed, a phenomenon frequently observed during field studies focussed on tidal trapping (MacVean & Stacey, 2011; Garcia et al., 2022). For example, Garcia et al. (2022) observed internal mixing in side creeks driven by a density current within the trap. By comparing their empirical estimate of tidal trapping with Eq. 2, they concluded that the observed internal mixing enhances the effect of tidal trapping. In the idealized model, the assumption of complete internal mixing significantly enhances the trap effect when $\alpha _d$ is low (see Fig. 10). However, when $\alpha _d > \frac{1}{4}\pi$, the effect of internal mixing becomes negligible. This occurs because the maximum particle separation due to internal mixing is achieved at $\alpha _d=0\pi$ and decreases for higher values, owing to the shape of the along-trap salinity profile.

#### Phase−Induced Diffusive Damping

For values of the Péclet number between $10^1$ and $10^3$, both diffusive and the cross-sectional average exchange was found to be important for the trap effect. Results from the numerical model indicate that when $\alpha _d$ is small, the diffusive exchange tends to increase the trap effect beyond what is expected from pure advective out-of-phase exchange (Fig. 10). However, when $\alpha _d$ is large and the trap is sufficiently long, a diffusive salt flux tends to diminish the trap effect. This occurs because the phase difference introduces a strong lateral gradient when the trap empties (Section “Transitions Between Advective Out-of-Phase and Diffusion Dominated Channel-Trap Exchange (Case 4)”), resulting in a diffusive salt flux into the trap, which reduces the sharpness of the resulting salt anomaly in the channel. To the authors’ knowledge, the diffusive damping of the trap effect, which results from diffusive salt flux into the trap and is set up by a sharp salinity gradient at the trap entrance during the ebb phase due to the flow velocity phase difference between the trap and the main channel, has not yet been documented in the literature.

#### Continuous Diffusive Exchange

When the contribution of advective exchange decreases ($P_e < 10^1$), tidal trapping results from a continuous diffusive exchange, which arises from a lateral salinity gradient between the channel and the trap, set up by the tidal motion. This type of exchange is often observed in real estuaries (Abraham et al., 1986; Ralston & Stacey, 2005; Giddings et al., 2012; De Nijs et al., 2009), especially near harbours. However, it should be noted that this type of exchange involves a density current flow, a mechanism not adequately captured within the 1D framework. The results indicate that when the trap is sufficiently long, the typical residence time matches the tidal cycle, and the trap effect is determined by the salt front’s propagation length. This makes it challenging to quantify the resulting trap effect using formulations like Eq. 1, which require specifying the relative trap size and residence time in advance.

### Development Towards a New Equilibrium

In Section “Results”, the additional salt flux was derived for a single cycle by adjusting the initial salinity field. However, as the system develops a new equilibrium, the exchange with the trap changes because of the adjusted along-channel salinity field.

**Fig. 12:**
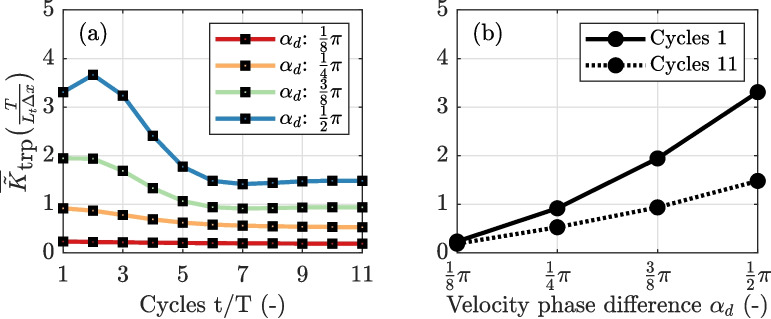
**a** Evolution of the excursion-averaged normalized dispersion coefficient $\frac{L_t \Delta x}{T}$ during the first ten tidal cycles, presented for various velocity phase differences. **b** Comparison of the excursion-averaged dispersion coefficient calculated at the start and after 11 cycles. Both are evaluated under the conditions of case 1

To explore the trap effect as the system develops towards an equilibrium, we quantified the additional salt flux over the first ten consecutive tidal cycles assuming pure advective out-of-phase exchange using Eq. 11, from which the excursion-averaged dispersion coefficient $\overline{\tilde{K}}_\text {trp}$ is calculated. The calculated values for $\overline{\tilde{K}}_\text {trp}$ show an initial decrease but stabilize after several tidal cycles (Fig. 12a). The model suggests that $\overline{\tilde{K}}_\text {trp}$ stabilizes when the tidally averaged salinity gradients within the tidal extent no longer show substantial deviations between consecutive tidal cycles. This suggests that the dispersion coefficient for tidal trapping depends on the salinity gradient, contrary to the common assumption that tidal trapping is independent of the salinity gradient (Okubo, 1973; Dronkers, 1978; Geyer & Signell, 1992). However, the dispersion coefficient is derived using the local $\frac{\partial s_0}{\partial x}$, which does not necessarily correspond to the same section where channel-trap exchange occurs and gradients are adjusted. This discrepancy leads to a dependency between the trap effect and the salinity gradient. To capture tide-averaged dispersion in a coefficient, the exchange should consistently relate to the mean gradient (Geyer & Signell, 1992). In the case of a local trap, the additional salt flux does not maintain a consistent relationship with the local mean gradient (Fig. 12).

**Fig. 13:**
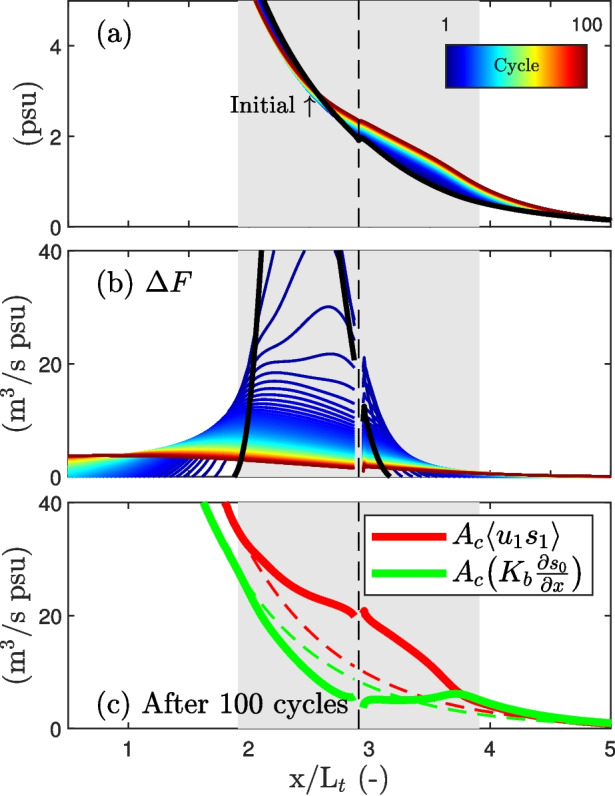
Development of the tidally averaged salinity distribution over 100 tidal cycles toward a new equilibrium, with $\alpha _d = \frac{1}{4}\pi$ and $K_b=20$ m$^2/s$ under case 1 channel-trap exchange conditions. The initial salt curve is shown by the solid black line. **a** Evolution of along-channel salinity distribution. In this example, the trap caused the 1 psu isohaline to migrate in up-estuary direction by 0.38$L_t$. **b** Salt flux difference $\Delta F = F- F_r$ relative to the reference simulation, with $\Delta F$ being equal to $F_\text {trp}$ in the first cycle. **c** The solid lines show the up-estuary salt fluxes in the new equilibrium after 100 cycles. The dashed lines show the salt fluxes for the equilibrium without the trap

A common assumption is that the effect of a trap is limited to the additional salt flux that directly results from channel-trap exchange. However, as the system evolves towards a new equilibrium, the total tidal salt flux attributable to the presence of the trap may differ significantly from the salt flux directly resulting from channel-trap exchange. To explain this, we recall that the tidal salt flux is defined as the difference between the vertical and lateral exchange observed by a Lagrangian plane and the exchange across a specific cross-section (Dronkers & Van de Kreeke, 1986). In a 1D setting, this simplifies to $A_{c} \langle u_1 s_1 \rangle = A_{c}K_b ( \frac{\partial \grave{s}_0}{\partial \grave{x}}- \frac{\partial s_0}{\partial x})$, where $\grave{()}$ indicates properties observed from the Lagrangian plane. The salinity gradients resulting from channel-trap exchange are advected in both the up- and down-estuary directions, influencing the tidal salt flux within the region from -$L_t$ to $L_t$ relative to the trap’s position $x_{h}$. This effect extends beyond the immediate location of direct exchange, affecting the overall tidal salt flux. This is further illustrated in Fig. 13, presenting the development of the system towards a new equilibrium. For the first tidal cycle, the additional salt flux $\Delta F = F-F_r$ equals the additional salt flux that directly results from channel-trap exchange. This initial local increase in the salt flux spreads and eventually results in an overall increase of the up-estuary salt flux as the system approaches an equilibrium (see Fig. 13b). After hundred cycles (close to a new equilibrium), the influence of the trap is evident in the tidal salt flux within the region from -$L_t$ and $L_t$ relative to $x_{h}$ (see Fig. 13c), exceeding the extent where the direct channel-trap exchange occurs.

**Fig. 14:**
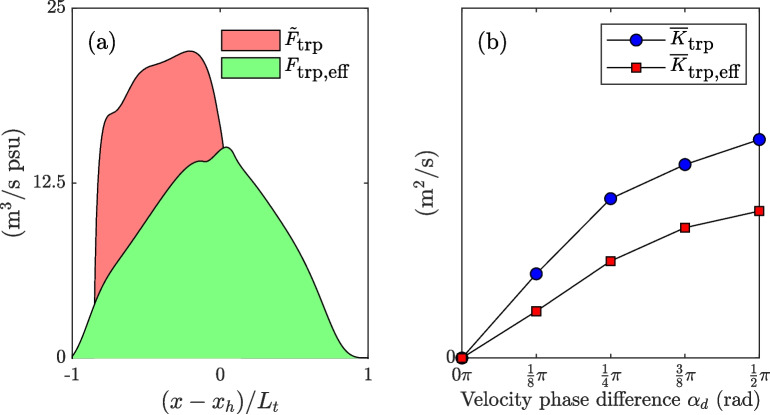
Relationship between direct mixing from channel-trap exchange and the resulting tidal salt flux associated with the trap. **a** Comparison between the salt flux directly resulting from channel-trap exchange and the overall observed increase in tidal salt flux associated with the trap from a simulation with $\alpha _d = \frac{1}{4}\pi$ and $K_b = 20$ m$^2/s$. **b** Comparison between the excursion-averaged additional dispersion ($\overline{K}_\text {trp}$) and the effective increase in tidal dispersion ($\overline{K}_\text {trp,eff}$) for different velocity phase differences ($\alpha _d$)

The increase in tidal salt flux is accompanied by a reduction in the diffusive salt flux, explained by the decreased gradients in the system. To highlight the difference between the salt flux directly resulting from the channel-trap exchange (quantified through Eq. 11) and the resulting increase in tidal salt flux in the region from -$L_t$ and $L_t$ relative to $x_{h}$, referred to as the effective trap effect ($F_\text {trp,eff}$), we compare these two quantities. For this comparison, we assume that the decay in tidal salt flux observed both up- and down-estuary outside the influence zone of the trap (resulting in a constant dispersion coefficient) continues toward the trap. Figure 14 compares the resulting dispersion coefficients, demonstrating that the dispersion coefficient attributed to the overall increase in tidal salt flux is lower than that resulting from direct channel-trap mixing alone. The impact of the trap occurs over a larger zone. This analysis underscores the challenge of quantifying the additional tidal salt flux associated with geometric features, such as dead-end side channels. Our results warrant caution when using analytical parametrizations of channel-trap exchange to interpret observations of tidal dispersion, particularly in the presence of substantial gradients in the along-channel salinity distribution.

## Conclusions

Tidal trapping, the temporal storage of water from the main channel in lateral dead zones and side channels, can increase the up-estuary salt transport. The exchange between channel and trap can occur through dispersive processes and advective out-of-phase exchange. Analytical frameworks typically assume one dominant exchange type to obtain solutions, but do not address the combined effect of both occurring simultaneously. Next, there exists an ambiguity in the literature regarding the dependence of the dispersive effect on the phase difference between the channel and the trap. This study quantifies the dispersive contribution from tidal trapping in a single dead-end side channel, accounting for both types of channel-trap exchange, using a 1D numerical model to quantify the additional salt flux resulting from different types of channel-trap exchange. The results show that pure advective out-of-phase exchange yields the largest additional salt flux for the greatest physically realistic velocity phase difference of 90$^{\circ }$. For small velocity phase differences between flow in trap and in the main channel junction region, mixing in the trap or continuous diffusive exchange enhances the trap effect. However, diffusive exchange can reduce the trap effect compared to pure advective out-of-phase exchange when the velocity phase difference is large. This is due to an enhanced diffusive salt flux that develops when the trap empties, lowering the salinity difference between the main channel and the re-discharged water from the trap (phase-induced diffusive damping). The additional salt flux in the main channel depends on the strength of background dispersion and the associated diffusive time-scales. Finally, the increase in tidal salt flux due to a trap in near-equilibrium conditions differs from the additional salt flux directly resulting from channel-trap exchange. This is because channel-trap exchange alters the salinity gradient over the region where the exchange occurs, equal to the tidal excursion length. The altered gradients are advected in both the up- and the down-estuary directions during the subsequent tidal cycle, influencing the resulting tidal salt flux over a distance twice the tidal excursion length.

## Figures and Tables

**Fig. 15:**
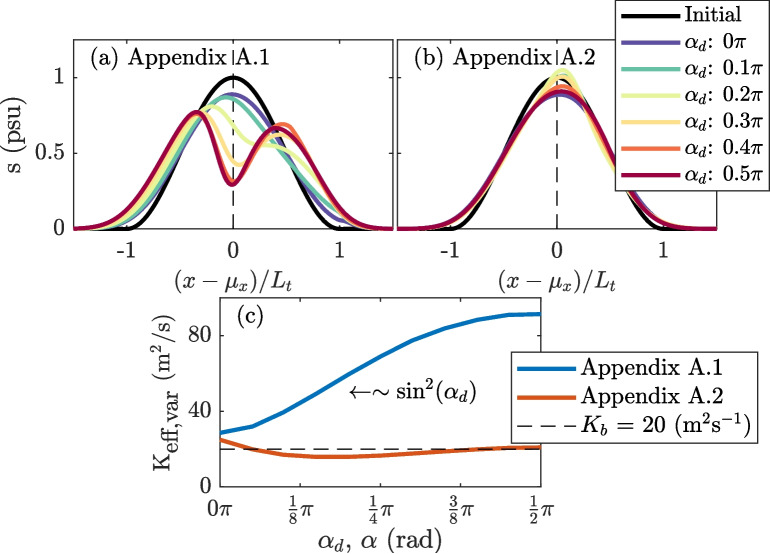
Resulting solute distributions and estimates of dispersion coefficients. **a** Initial solute distribution and the resulting distribution after one tidal cycle for pure advective out-of-phase exchange (see Section “A.1.    An Initial Pulse of Solutes Subjected to Pure Out-of-Phase Tidal Trapping”). The x-axis shows the distance from the centre of the distribution $\mu _x$, normalized by the tidal excursion length $L_t$. **b** Same as **a**, but under the assumption of a uniform along-channel velocity (see Section “A.2.    Assuming a Uniform Main Channel Velocity”). **c** The effective dispersion coefficient $K_\text {eff,var}$ for both cases, determined from the resulting solute distributions using Eq. A1, as a function of the velocity phase difference $\alpha _d$ and $\alpha$

**Fig. 16:**
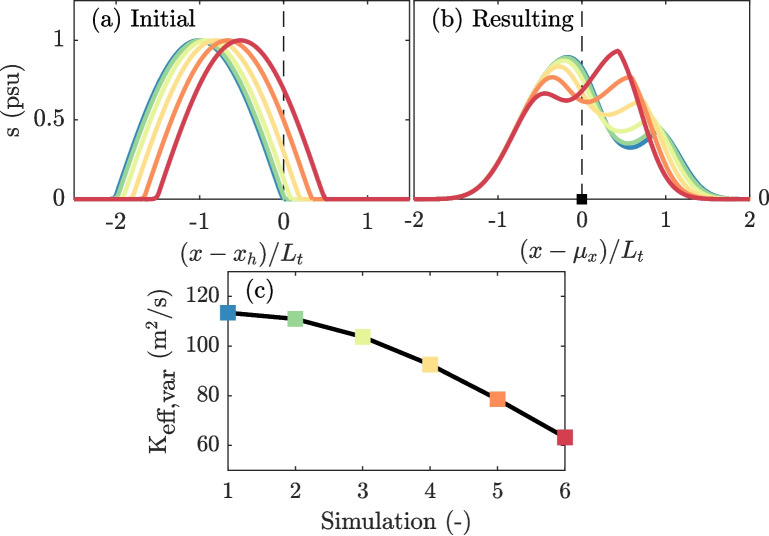
Illustration of the dependency of $K_\text {eff,var}$ on the initial solute distribution when adopting a non-generalized source-sink term. **a** Assumed initial solute distribution. **b** Resulting solute distribution for pure advectivive out-of-phase exchange with the velocity in trap and channel in phase ($\alpha = 0$), but assuming a non-generalized source-sink term. Here, for all simulation, the concentration at the trap entrance is given by A8a, with $\hat{S}_t =1$ and $\alpha =0$. **c** The effective dispersion coefficient $K_\text {eff,var}$ determined from the resulting solute distributions using Eq. A1, plotted as a function of the assumed initial condition

**Fig. 17:**
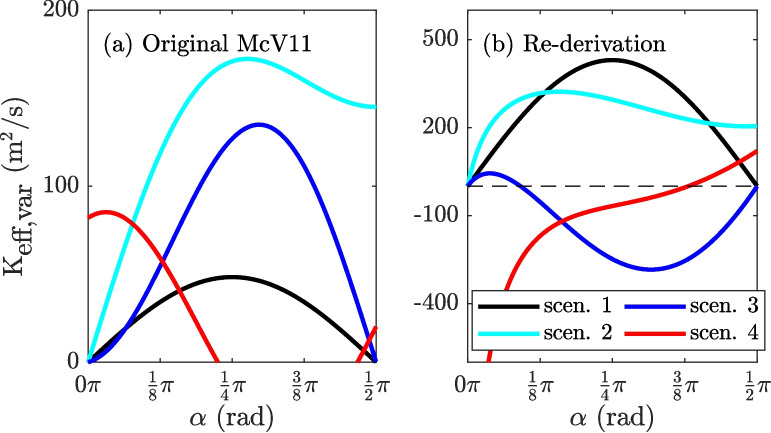
Comparison of the dispersion coefficient $K_{\text {eff,var}}$ according to the analytical solution presented by MacVean and Stacey (2011) and the results from the re-analysis. **a** The theoretical curves for the different scenarios analyzed by MacVean and Stacey (2011). **b** The curves obtained after the re-derivation (see Eqs. A9a–d)

**Fig. 18:**
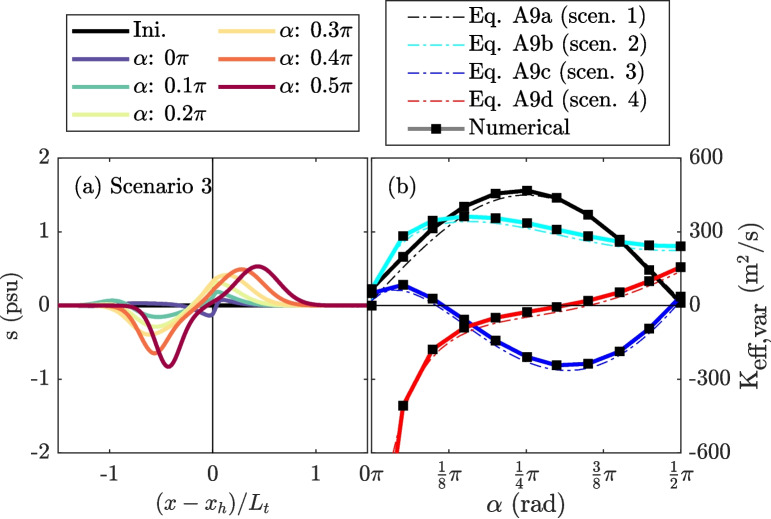
**a** Illustration of the resulting solute distribution after one tidal cycle for scenario 3 (see Section “A.3.3.    Validation Through Numerical Experiments”). **b** Comparison of the effective dispersion coefficient, $K_\text {eff,var}$, calculated from numerically determined solute distributions (using Eq. A5) and the analytical expressions from Eq. A9, as a function of the velocity phase difference, $\alpha$

## Data Availability

The data and code used to generate the figures are available at: https://doi.org/10.5281/zenodo.14905063.

## Appendix A. Explanation of Differences with MacVean and Stacey (2011)

The dependence of salinity dispersion on the velocity phase difference between a main channel and a trap, as established in this study, aligns with the findings of Dronkers (1978), but stands in contrast to those reported by a more recent paper by MacVean and Stacey (2011). To understand the latter contrast, here we identify several key differences in the model assumptions and the methods to quantify the trap effect. We systematically assess the influence of each of these assumptions, which is supported by a series of numerical experiments.

Appendix A.1 investigates the possible differences that proceed from assuming an initial solute pulse as assumed in the concentration moment analysis (Aris, 1956; Fischer et al., 1979), which is a starting point for MacVean and Stacey (2011). Appendix A.2 highlights the importance of the velocity difference between the upstream and downstream reaches relative to the trap in quantifying the dispersive effect of advective out-of-phase exchange. The latter is not accounted for in MacVean and Stacey (2011). The contrasting assumptions explained in Appendices A.1 and A.2 do not fully explain the observed differences. Even after adopting the assumptions of MacVean and Stacey (2011) regarding initial concentration, source-sink term, and definition of dispersive effect, consistent agreement between analytical and numerical results was only achieved after refining the analytical derivation in MacVean and Stacey (2011), which is elaborated in the Appendix A.3. A summary of our findings explaining the discrepancy is provided in Appendix A.4.

### A.1. An Initial Pulse of Solutes Subjected to Pure Out-of-Phase Tidal Trapping

A difference between this study and MacVean and Stacey (2011) lies in how the trap effect is determined. In this study, we adopted an initial salinity distribution that decreases monotonically in the up-estuary direction and quantify the trap-induced additional salt flux through a cross-section (Section “Analysis of Model Results”). MacVean and Stacey (2011) considered the evolution of an initial solute pulse and relate the effective dispersion to the rate of increase of the variance of the pulse. Following the approach of MacVean and Stacey (2011), we here consider the evolution of a solute pulse and express the effective dispersion using the concentration moment analysis (Aris, 1956; Fischer et al., 1979):

A1 $$\begin{aligned} K_\text {eff,var} = \frac{1}{2} \frac{\partial \sigma ^2}{\partial t} = \frac{\partial }{\partial t} \biggl ( \frac{M_2}{M_0} - \mu _x^2 \biggr ), \end{aligned}$$

where $$\sigma ^2$$ is the variance of the solute distribution, $$M_0$$ is the zeroth moment, $$M_2$$ is the second moment, and $$\mu _x = \frac{M_1}{M_0}$$ is the location of the centroid of the solute distribution. The $$\textit{i}$$-th raw moment $$ M_{i}$$ of the solute concentration *s*(*x*, *t*) is defined as

A2 $$\begin{aligned} M_{i} = \int _{- \infty }^{\infty } x^i s(x,t) dx. \end{aligned}$$

The variance $\sigma ^2$ can be expressed using the moments as follows:

A3 $$\begin{aligned} \sigma ^2 = \frac{\int _{- \infty }^{\infty } (x-\mu _x)^i s(x,t) dx}{ M_0} = \bigg (\frac{M_2}{M_0}\bigg )-\mu _x^2 . \end{aligned}$$

Since we are interested in the change in the variance after one full tidal cycle, we define $K_\text {eff,var} = \frac{1}{2T} \big (\sigma ^2|_{t=T}-\sigma ^2|_{t=0} \big )$ to express the dispersive effect of the trap.

To evaluate the evolution of an initial solute distribution, we set up a numerical model for pure advective out-of-phase exchange, in which the initial salinity distribution in the main channel is defined as

A4 $$\begin{aligned} s(x-x_h,0) = \left\{ \begin{array}{ll} \frac{1}{2}\big ( 1\!-\! \cos (k(x-x_h)-\lambda )\big ) & \text {if } \big (\frac{\lambda }{\pi T}-2\big ) L_t\\ \le \big (x-x_h\big ) \le \frac{L_t\lambda }{\pi T}; \\ 0 & \text {otherwise}, \end{array}\right.  \end{aligned}$$

with $k=\frac{\pi }{L_t}$ and $\lambda =\frac{\pi }{2} \big ( 1- \cos (-\alpha )\big )$. This initial salinity profile enables quantification of $K_\text {eff,var}$ through Eq. A1.

Figure 15a presents the initial and resulting solute distributions after one tidal cycle for different values of $\alpha _d$. The corresponding values of $K_\text {eff,var}$, quantified using Eq. A1, are shown in Fig. 15c. The plot shows $K_\text {eff,var}$ scales with $\sin ^2(\alpha _d)$, with the trap effect reaching its maximum at $\alpha _d = \frac{1}{2}\pi$. Based on this experiment, we conclude that the differences with MacVean and Stacey (2011) cannot directly be attributed to differences in the initial salinity distribution or to the method used to quantify the trapping contribution. Note that at $\alpha _d = 0$, the values of $K_\text {eff,var}$ are not zero. Then, it is close to the value used for $K_b$.

### A.2. Assuming a Uniform Main Channel Velocity

A second key difference concerns the velocity in the main channel. MacVean and Stacey (2011) used a uniform along-channel velocity, whereas in this study, a difference between the channel velocity up-estuary and down-estuary of the trap is introduced. This difference is the result of the presence of the trap and is calculated using the continuity equation. Note that a uniform along-channel velocity was adopted by Okubo (1973) to evaluate a continuous lateral trap where the exchange is driven by diffusive processes. However, in a trap where the exchange is driven by advective processes, a velocity phase difference is introduced between the parts of the main channel up-estuary and down-estuary of the trap, leading to a dispersive effect as discussed in Section “Changes in the Along-Channel Salinity Profile (Case 1)”.

We conducted a second set of experiments, assuming a uniform velocity in the main channel, consistent with the approach of MacVean and Stacey (2011). The initial salinity profile is again given by Eq. A4, and for the source-sink term, pure advective out-of-phase exchange is assumed (see Fig. 15b,c). In contrast to the experiment in Appendix A.1, the variance of the solute distributions does not increase with an increasing velocity phase difference. Instead, it exhibits a slight decrease and is most pronounced for $\alpha _d \approx \frac{1}{4}\pi$. This experiment emphasizes the importance of the along-channel non-uniform velocity in quantifying the effect of advective out-of-phase exchange and clearly demonstrates that its dispersive effect cannot be accurately described unless the along-channel velocity difference introduced by the trap is taken into account.

### A.3. Reproducing the Solution by MacVean and Stacey (2011)

Sections “A.1.    An Initial Pulse of Solutes Subjected to Pure Out-of-Phase Tidal Trapping” and “A.2.    Assuming a Uniform Main Channel Velocity” do not fully explain the differences between results presented in this paper and those by MacVean and Stacey (2011). Two key assumptions adopted by MacVean and Stacey (2011) to obtain an analytical expression remain unexplored: (1) the relationship between salinity in the main channel and the salinity level at the trap entrance, which determines the concentration of salt abstracted from (or released into) the main channel by the source-sink term, and (2) the adopted definition of $K_\text {eff,var}$. Those assumptions are discussed in Appendix A.3.1. After adopting the assumptions by MacVean and Stacey (2011), Appendices A.3.2 and A.3.3 seek to find consistent results between the analytical expressions and numerical model results, which is only achieved for an adjusted analytical expression.

#### A.3.1. The Adopted Source-Sink Term and Definition of the Dispersive Effect

The concentration moment analysis developed by Aris (1956), adopted by MacVean and Stacey (2011), provides a more general solution to Taylor’s problem of shear dispersion (Taylor, 1953), as it does not require explicit specification of the salinity distribution in the main channel (Aris, 1956; Fischer et al., 1979). MacVean and Stacey (2011) assumed that this generality also applies to their model. However, their approach includes a source-sink term that removes solutes from the channel at a predefined concentration. This assumption introduces a dependency on the initial conditions, thereby reducing the generality of the results. In contrast, Okubo (1973) employed a generalized source-sink formulation in which both the source and sink terms are functions of the salinity in the main channel. This approach preserves the generality of Aris (1956) method. To illustrate that $K_\text {eff,var}$ depends on the initial conditions when a predefined source-sink term is used, we performed a series of model runs—assuming a uniform main channel velocity—in which the system was initialized with a solute distribution described by Eq. A4. Herein, $\alpha$ is varied between 0 and $\frac{1}{2}\pi$. We employed the source-sink term adopted by MacVean and Stacey (2011) for pure advective out-of-phase exchange (see Eq. A8c), but assumed that the velocity in the trap is in phase with the main channel. Figure 16 illustrates the resulting solute distributions and the corresponding values of $K_\text {eff,var}$, quantified through Eq. 6.1, which clearly vary depending on the assumed initial condition.

MacVean and Stacey (2011) do not specify an initial salinity distribution in the main channel, but prescribe a salinity at the trap entrance that fluctuates around zero to define the source-sink term. As a result, the initial concentration in the main channel can be interpreted as zero. The solute distribution that develops in the main channel after one full tidal cycle is therefore solely the outcome of the source-sink dynamics and includes both positive and negative concentration values. If there is no net salt flux from the trap (as is the case for advective out-of-phase exchange), then $M_0$ remains equal to zero after a full tidal cycle. This presents a problem when attempting to determine the variance of the distribution using Eq. A3. Instead, MacVean and Stacey (2011) defined $K_\text {eff,var}$ as follows:

A5 $$\begin{aligned} K_\text {eff,var} = \frac{1}{2} \frac{\partial \sigma ^2}{\partial t} =\frac{1}{2T} \frac{\Delta M_2}{\overline{M_0}}, \end{aligned}$$

where it is assumed that $\mu _x = 0$ and $M_0$ is replaced by the tidal averaged $\overline{M_0}$. Setting $\mu _x = 0$ means that the centre of the distribution is always assumed to coincide with $x = 0$. In their analytical derivation, MacVean and Stacey (2011) take $x = 0$ to coincide with the position of the trap. This can be questioned, especially in cases where there is a net salt flux from the trap. Before numerically evaluating the effect of no initial concentration with a predefined source-sink term (Section “A.3.3.    Validation Through Numerical Experiments”), we will first re-examine the four analytical solutions presented by MacVean and Stacey (2011) in the next section.

#### A.3.2. Re-Derivation of Analytical Expressions

The salt balance assumed by MacVean and Stacey (2011) reads:

A6 $$\begin{aligned} \frac{\partial s}{ \partial t} + \hat{U}_c\sin (\omega t-\alpha )\frac{\partial s}{\partial x} -\frac{\partial }{\partial x}\biggl ( K_b\frac{\partial s}{ \partial x} \biggr ) = -\frac{\hat{U}_t H_t}{A_c} \sin (\omega t) \end{aligned}$$

Differentiating Eq. 6.2 with respect to time, substitution of this salt balance into the obtained relation, and integration over time yield expressions for the concentration moments. Substituting those into Eq. A5 leads to a general expression for $K_\text {eff,var}$:

A7 $$\begin{aligned} K_\text {eff,var}&= -\frac{\hat{U}_c^2 \hat{Q}_T}{\overline{M_0} T A_c} \int _0^T \sin (\omega t-\alpha )\nonumber \\&\times \bigg (\int _0^t \sin (\omega t-\alpha ) \bigg ( \int _0^t \sin (\omega t)s_t(t) dt\bigg ) dt\bigg )dt\nonumber \\&- \frac{l^2 \hat{Q}_T}{6 \overline{M_0} T A_c}\int _0^T \sin (\omega t)s_t(t) dt + K_ b, \nonumber \\ \end{aligned}$$

where $l = \frac{1}{2}\Delta x$ and $s_t(t)$ is the salinity at the trap entrance. The four analytical expressions presented in MacVean and Stacey (2011) are derived by adopting the following functional forms for $s_t(t)$:

A8a $$\begin{aligned} s_t(t)_{\text {scen. 1}}&= -\hat{S}_t \cos (\omega t),\end{aligned}$$

A8b $$\begin{aligned} s_t(t)_{\text {scen. 2}}&= -\hat{S}_t \cos (\omega t- \alpha ),\end{aligned}$$

A8c $$\begin{aligned} s_t(t)_{\text {scen. 3}}&= {\left\{ \begin{array}{ll} -\hat{S}_t \cos (\omega t- \alpha ) & 0< t< T/2; \\ \hat{S}_t \cos (\omega \big (\frac{T}{2}- t\big )- \alpha ) & T/2< t < T, \end{array}\right. }\end{aligned}$$

A8d $$\begin{aligned} s_t(t)_{\text {scen. 4}}&= {\left\{ \begin{array}{ll} -\hat{S}_t \cos (\omega t- \alpha ) & 0< t< T/2; \\ \frac{1}{T}\int _0^{T/2} -\hat{S}_t \cos (\omega t- \alpha )dt & T/2< t < T, \end{array}\right. } \end{aligned}$$

where $\hat{S}_t$ denotes the salinity amplitude at the trap entrance. The subscript $\text {scen.}$, scenario, corresponds to the cases considered by MacVean and Stacey (2011). We use the term scenario instead of case here to distinguish them from the case studies introduced in main text of this paper. We are unable to reproduce the expressions presented by MacVean and Stacey (2011) when using the functional forms given in Eq. A8 to obtain an explicit solutions to Eq. A7. Instead, we find the following four expressions (see the accompanying MATLAB code for the derivation):

A9a $$\begin{aligned} K_\text {eff, scen. 1}&= \hat{U}_cL_t \frac{\cos (\alpha ) \sin (\alpha ) }{4 } +K_b ,\end{aligned}$$

A9b $$\begin{aligned} K_\text {eff, scen. 2}&= \hat{U}_cL_t \sin (\alpha ) \frac{7- 4\sin ^2(\alpha ) }{16\sin (\alpha )\pi + 8\cos (\alpha )}\nonumber \\ &- \frac{2l^2 \pi \sin (\alpha )}{3T(\cos (\alpha )+2\pi \sin (\alpha ))} +K_b, \end{aligned}$$

A9c $$\begin{aligned} K_\text {eff, scen. 3}&= \hat{U}_cL_t \cos (\alpha ) \frac{(32\cos (\alpha )^2 \!+\! 3\pi \cos (\alpha )\sin (\alpha ) \!-\! 32)}{(12\pi (\cos (\alpha ) + \pi \sin (\alpha ))} +K_b.\end{aligned}$$

A9d $$\begin{aligned} K_\text {eff, scen. 4}&= \hat{U}_cL_t \Biggl [ \frac{ \big (16-9\pi ^2\big )\sin (\alpha )}{24\pi \big ( 3\sin (\alpha )\pi ^2+\cos (\alpha )\pi -2\sin (\alpha ) \big )}\nonumber \\&+\frac{\big ( 16-12\pi ^2 \big ) \cos (\alpha )^2\sin (\alpha ) }{24\pi \big ( 3\sin (\alpha )\pi ^2+\cos (\alpha )\pi -2\sin (\alpha ) \big )}\nonumber \\ &+ \frac{76\pi \cos (\alpha ) - 44\pi \cos (\alpha )}{24\pi \big ( 3\sin (\alpha )\pi ^2+\cos (\alpha )\pi -2\sin (\alpha ) \big )} \nonumber \\&\quad \!-\! \frac{2}{3\pi }\frac{l^2}{L^2} \sin (\alpha ) \frac{\pi ^2-2}{3\sin (\alpha )\pi ^2+\cos (\alpha )\pi -2\sin (\alpha )} \Biggr ] \!+\! K_b. \end{aligned}$$

Beyond the analytical differences, a key distinction is that Eqs. A9a–d do not include the non-dimensional ratio $\epsilon = \frac{\hat{S}_t }{\hat{S}} \frac{\hat{Q}_t}{\hat{Q}_c} = \frac{\hat{S}_t }{\hat{S}} \frac{A_t}{A_c}\frac{\hat{U}_t}{\hat{U}_c}$, which appears in the formulations presented by MacVean and Stacey (2011). This ratio emerges when adopting the same scaling assumptions used in their analysis, specifically assuming that $\overline{M_0}$ scales as $\hat{S} L_t$. Under this scaling assumption, the parameters in front of the integral term in Eq. A7 (after taking $\hat{S}_t$ out of the integral) can be rewritten as $\frac{\hat{U}_c^2 \hat{Q}_T \hat{S}_t}{\overline{M_0} T A_c} \sim \epsilon \hat{U}_cL_t$. However, this essentially implies that we define $K_{\text {eff,var}} = \frac{1}{2T} \frac{\Delta M_2}{\overline{M_0}} \sim \frac{1}{2T} \frac{\Delta M_2}{L_t \hat{S}}$. This scaling approach introduces an inconsistency with the derivation of $\Delta M_2$ and affects $K_b$ in Eq. A7. Adopting this scaling approach leads to the following expression for scenario 3:

A10 $$\begin{aligned} K_\text {eff, scen. 3} =&\epsilon \hat{U}_cL_t \cos (\alpha ) \frac{(32\cos (\alpha )^2 + \frac{3}{2}\pi \sin (\alpha ) - 32)}{96\pi }\nonumber \\&+\epsilon K_b\frac{\cos (\alpha )+\pi \sin (\alpha )}{8}. \end{aligned}$$

Equation A10 is similar to the equation presented by MacVean and Stacey (2011) (see Eq. 2), especially when we express $L_t = \frac{2\hat{U}_c}{\omega }$.

Figure 17 compares the resulting curves from Eq. A9 with the results presented by MacVean and Stacey (2011). The shape of the curves resulting from cases 1 and 2 is similar. For case 3 (representing pure advective out-of-phase exchange), the new equations predict a similar shape, but result in negative dispersion coefficients. For case 4 (complete mixing of the trapped salinity field), we obtain a curve that differs significantly from the one presented by MacVean and Stacey (2011).

#### A.3.3. Validation Through Numerical Experiments

To test whether we can numerically reproduce the newly derived analytical expressions Eqs. A9a–d, we initialize the model with no solute present in the domain, i.e. $s(x, 0) = 0$, and adopt the source-sink term formulation used by MacVean and Stacey (2011). Furthermore, we assume a constant velocity in the main channel, as done in Section “A.2.    Assuming a Uniform Main Channel Velocity”, and quantify $K_\text {eff,var}$ using Eq. A5. Figure 18a illustrates the resulting salinity distribution for case 3 (advective out-of-phase exchange), and Fig. 18b compares the numerical results for the different scenarios with Eqs. A9a–d. The numerical solutions confirm each of the four analytical solutions in Eqs. A9a–d.

### A.4. Concluding Remarks

The analytical expressions presented by MacVean and Stacey (2011) do not capture the dispersive effect of advective out-of-phase exchange. We identified the following aspects where improvements are necessary: (1) account for the difference in along-channel velocity upstream and downstream of the trap, as this is an essential feature that determines the dispersive effect of advective out-of-phase exchange. (2) Adopt a generalized source-sink term to derive an expression for the dispersive effect using concentration moment analysis without requiring precise specification of the initial solute distribution (Aris, 1956). (3) Corrections are necessary in the derivation of the analytical expressions for the different cases (see Section “A.3.2.    Re-Derivation of Analytical Expressions”). Altogether, these aspects explain the observed differences in how the trap effect depends on the velocity phase difference between MacVean and Stacey (2011) and the present study. In this study, the model and analysis we adopt account for these aspects and therefore provide an improved and more general framework for describing dispersion due to tidal trapping by advective out-of-phase exchange.
